# Metabolic syndrome in colorectal cancer liver metastasis: metabolic reprogramming and microenvironment crosstalk

**DOI:** 10.3389/fimmu.2025.1653442

**Published:** 2025-10-14

**Authors:** Zheng Ma, Song Wang, Shanglong Liu, Wenchang Yang, Jilin Hu, Lianghong Lv, Qian Yu, Yun Lu

**Affiliations:** ^1^ Department of Gastrointestinal Surgery, The Affiliated Hospital of Qingdao University, Qingdao, China; ^2^ Tumor Immunology and Cytotherapy of Medical Research Center, Shandong Provincial Key Laboratory of Clinical Research for Pancreatic Diseases, the Affiliated Hospital of Qingdao University, Qingdao University, Qingdao, China

**Keywords:** metabolic syndrome, colorectal cancer liver metastasis, metabolic reprogramming, metabolomics, tumor microenvironment

## Abstract

Colorectal cancer (CRC) ranks as the third most frequently occurring cancer worldwide and the second major contributor to tumor-related mortality, frequently metastasizes to the liver due to its unique vascular and anatomical features, making liver metastasis a critical therapeutic challenge. Metabolic syndrome (MS) is a cluster of conditions characterized by insulin resistance as the core feature, specifically manifesting as obesity, diabetes, hypertension and dyslipidemia, exacerbates CRC progression through multifaceted mechanisms. MS induces mitochondrial dysfunction, oxidative stress, chronic inflammation, and Deoxyribonucleic Acid (DNA) methylation abnormalities, collectively promoting tumor cell proliferation and invasion. In the liver microenvironment, MS-driven metabolic disturbances foster fatty liver formation, alter pH via hyperglycemia, and enhance tumor energy supply. Pro-inflammatory factors and oxidative stress damage hepatocytes and endothelial cells, while immune dysregulation facilitates tumor immune escape. Angiogenic abnormalities further support metastatic growth. Recent studies highlight the strong correlation between metabolic disorders and colorectal cancer liver metastasis (CRLM). Metabolomics has emerged as a pivotal tool for identifying novel biomarkers, offering insights for early diagnosis and prognosis optimization. This article aims to review the metabolic changes in CRLM by exploring metabolic reprogramming, the role of MS in driving CRLM, and the critical importance of metabolomics in CRLM. By providing scientific evidence, this review seeks to identify novel therapeutic targets and develop personalized treatment strategies for patients with CRLM. Furthermore, it aims to further advance the in-depth exploration of CRLM-related mechanisms and promote the rapid development of clinical translation.

## 1 Introduction

Colorectal cancer (CRC) ranks among the most common malignant neoplasms globally, with its incidence and mortality rates placing third and second, respectively (1). The high mortality rate of CRC is primarily attributed to its propensity for distant metastasis, particularly to the liver. As the most frequent site of metastasis for CRC, approximately 50% of CRC patients develop colorectal cancer liver metastasis (CRLM) during the disease. CRLM substantially reduces the five-year survival rate of patients and poses significant challenges to clinical management (2). In recent years, with the rapidly increasing prevalence of metabolic syndrome (MS), mounting evidence suggests a close association between MS and the onset, progression, and metastasis of CRC (3). However, the precise mechanisms by which MS promotes CRLM through complex metabolic reprogramming, microenvironmental regulation, and molecular pathways remain incompletely elucidated.

MS encompasses a cluster of metabolic disorders characterized by obesity, diabetes, hypertension, and dyslipidemia. These pathological conditions not only induce systemic metabolic dysregulation but also influence the tumor microenvironment (TME) through various pathways, thereby enhancing tumor cell proliferation, invasion and metastasis. Chronic inflammation induced by MS activates signaling pathways such as NF-κB, STAT3, and PI3K/AKT, thereby augmenting the malignant phenotype of tumor cells (4). For instance, elevated insulin levels can stimulate cell proliferation via the insulin-like growth factor-1 (IGF-1) axis while inducing epithelial-mesenchymal transition (EMT), thereby enhancing tumor invasiveness (5). Furthermore, lipid metabolism dysregulation associated with MS, such as enhanced fatty acid oxidation, provides tumor cells with additional energy sources, supporting their rapid proliferation within the hepatic microenvironment. MS also contributes to CRLM by inducing mitochondrial dysfunction, oxidative stress, and aberrant epigenetic modifications, such as altered DNA methylation. Research indicates that oxidative stress, prevalent in patients with metabolic syndrome, can induce DNA damage and genomic instability, thereby promoting oncogene activation and tumor suppressor gene inactivation (6). Moreover, aberrant DNA methylation and histone modifications are particularly pronounced in CRC patients with MS. These epigenetic alterations may reshape tumor cell metabolism by modulating the expression of key metabolic enzymes (7). In CRLM, these metabolic aberrations interact with the liver’s unique anatomical structure and vascular properties, creating a “fertile soil” that supports tumor growth and metastasis (8). For instance, elevated glucose and fatty acid levels in the hepatic microenvironment can fuel metastatic tumor cells by providing energy and biosynthetic precursors through the Warburg effect and lipid metabolism reprogramming (8).

In recent years, metabolomics, an emerging systems biology tool, has demonstrated significant potential in comprehensively characterizing metabolites within tumors and their microenvironments through high-throughput techniques. This approach not only aids in elucidating tumor metabolic profiles and identifying potential biomarkers but also offers novel insights into the metabolic characteristics of CRLM (9). In studies of MS-associated CRLM, metabolomics has not only elucidated the molecular mechanisms of metabolic reprogramming but also identified potential biomarkers for early diagnosis and personalized therapy. For instance, multi-omics integrative analyses based on metabolomics have successfully pinpointed metabolic signatures linked to CRLM prognosis, such as disruptions in phospholipid metabolism and amino acid metabolism abnormalities (10). Furthermore, the integration of metabolomics with machine learning techniques enables the development of risk prediction models, significantly enhancing the diagnostic accuracy and reliability of treatment response evaluation for CRLM. In the future, targeting critical metabolic pathways, such as fatty acid synthesis and glutamine metabolism, holds promise for developing novel therapeutic strategies, offering precision medicine solutions for CRLM patients.

This article aims to review the metabolic alterations in CRLM, with a focus on exploring metabolic reprogramming in CRLM, the role of MS in driving CRLM, and the latest advancements in metabolomics research in this field. By integrating basic research with clinical practice, we anticipate significantly improving the prognosis of CRLM patients in the future, paving new avenues for precision medicine in metabolism-related tumors, and further advancing the in-depth exploration of CRLM-related mechanisms and the rapid development of clinical translation.

## 2 The metabolic processes driving the onset and advancement of colorectal cancer liver metastasis

**Figure 1:**
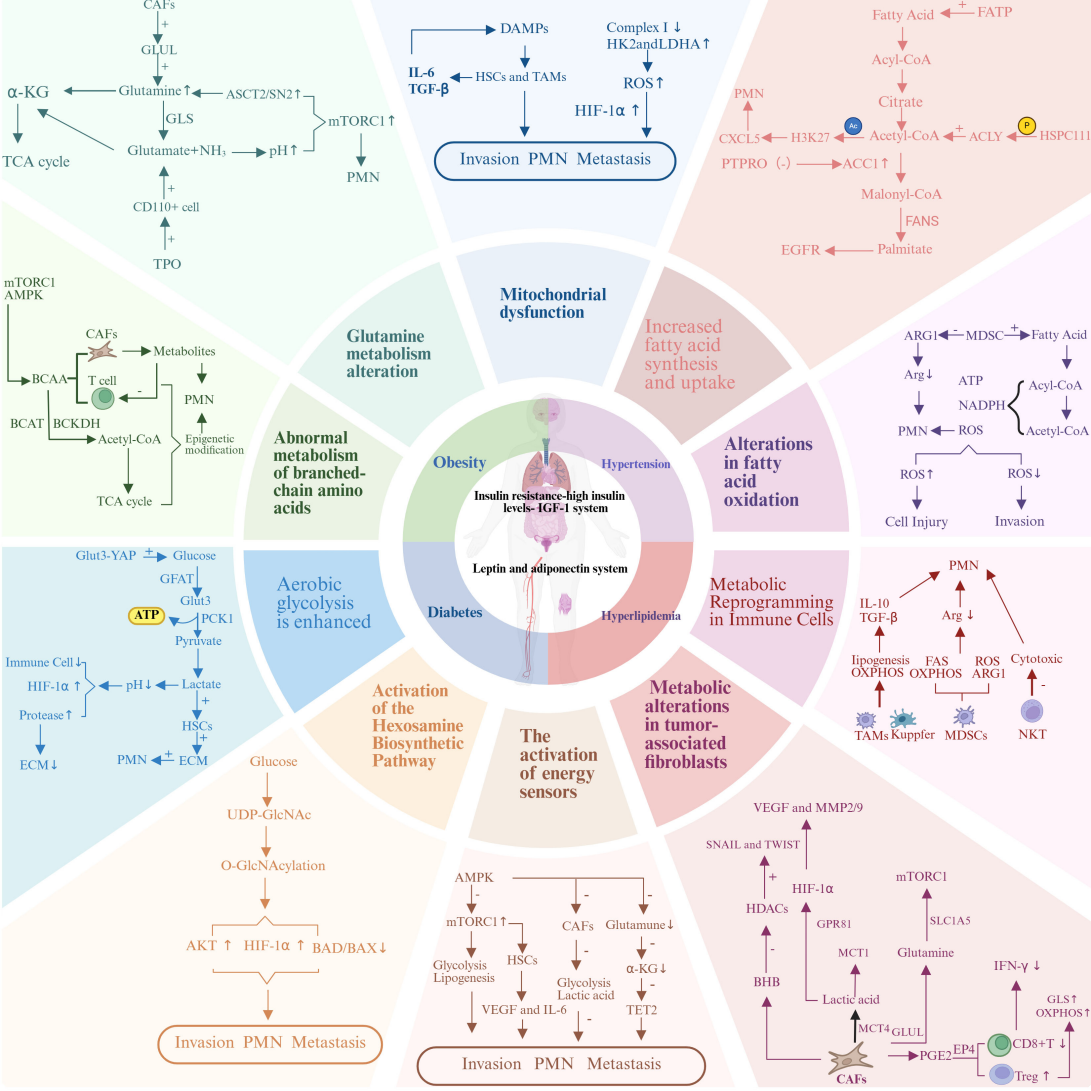
Metabolic reprogramming in colorectal cancer liver metastasis. The relationship between the metabolic dual axis (insulin-adipokine axis) in colorectal cancer liver metastasis and the core components of metabolic syndrome (obesity, diabetes, hyperlipidemia, and hypertension) with metabolic reprogramming.

Metabolic reprogramming is a central driver of the initiation and progression of CRLM, enabling tumor cells to adapt their metabolic pathways to meet the energy and biosynthetic demands required for rapid proliferation, invasion, and metastasis. Critically, this metabolic reprogramming follows a defined sequence. It starts with the formation of pre-metastatic niche (PMN) in the liver. These niches are specialized microenvironments. Primary tumor–derived factors and systemic metabolic changes linked to metabolic syndrome condition these niches. These changes create favorable conditions for metastatic tumor cells to arrive, survive, and colonize the liver (11). The liver’s unique metabolic microenvironment provides optimal metabolic support for CRLM. The establishment of PMN is a critical early step in metastasis. Primary colorectal tumors release soluble factors, exosomes, and metabolites. These signals travel through the bloodstream to reach the liver. They condition the liver microenvironment even before cancer cells arrive. This conditioning recruits bone marrow–derived cells (BMDCs). It also activates hepatic stellate cells (HSCs) and reprograms the metabolism of resident liver cells. Together, these changes create an environment that supports metastasis (12). At the center of this process are metabolic alterations within both tumor cells and the surrounding liver microenvironment. Collectively termed metabolic reprogramming, these changes allow tumor cells to fulfill their bioenergetic and biosynthetic needs, evade host defenses, and establish metastatic niches. Metabolic reprogramming in CRLM involves dysregulation of critical pathways, including lipid metabolism, glucose metabolism, amino acid metabolism, energy homeostasis, and microenvironmental metabolism (**Figure 1**).

### 2.1 Abnormal lipid metabolism

Lipid metabolism dysregulation is a critical driver in the initiation and progression of CRLM. Tumor-secreted factors from primary colorectal cancers induce systematic changes in hepatic lipid metabolism, which precede and enable tumor cell colonization. These tumors release lipid-enriched exosomes, lysophosphatidic acid (LPA), and other bioactive lipids into the bloodstream, where they target the liver. Upon reaching the liver, these lipid mediators activate HSCs and recruit BMDCs, initiating the formation of pre-metastatic niches (12). These tumor-derived lipids trigger metabolic reprogramming in both hepatocytes and stromal cells, redirecting their lipid metabolism toward the synthesis and secretion of compounds that establish a lipid-rich microenvironment, conducive to metastatic colonization. As the primary organ for lipid metabolism, the liver provides a lipid-rich microenvironment that serves as an optimal source of energy and biosynthetic precursors for metastatic tumor cells (13). Metabolic syndrome plays a crucial role in amplifying pre-metastatic niche formation, primarily through lipid dysregulation. This includes increased levels of circulating free fatty acids, disrupted cholesterol metabolism, and heightened lipogenesis (14). These metabolic changes contribute additional conditioning factors that activate HSCs, thereby creating a more favorable environment for the establishment of pre-metastatic niches. Under normal physiological conditions, lipid metabolism is tightly regulated to maintain energy homeostasis and support cellular functions. However, in the context of cancer, tumor cells often undergo metabolic reprogramming characterized by increased lipogenesis, lipid uptake, and altered fatty acid oxidation. The pre-metastatic conditioning of the liver involves a coordinated shift in lipid metabolism across various cell types, including hepatocytes, HSCs, Kupffer cells, and infiltrating myeloid cells. Each of these cells undergoes metabolic reprogramming in response to signals from primary tumors (14). These alterations not only supply essential building blocks for rapid cell proliferation but also contribute to the establishment of a tumor-supportive microenvironment in the liver. Therefore, a comprehensive understanding of the molecular mechanisms underlying lipid metabolism dysregulation can elucidate the metabolic reprogramming in CRLM and provide a theoretical foundation for developing therapeutic strategies targeting lipid metabolism.

#### 2.1.1 Increased fatty acid synthesis and uptake

Dysregulation of lipid metabolism has been established as a critical metabolic characteristic of cancer (15, 16). Studies suggest that numerous enzymes associated with fatty acid absorption, production, and breakdown contribute to the aberrant regulation of various cancers, resulting in fatty acid metabolism rewiring and fostering the malignant characteristics of cancer (17, 18). The synthesis of fatty acids from scratch represents a pivotal feature of metabolic reprogramming in malignant cells. In healthy cells, *de novo* fatty acid synthesis (FAS) largely depends on extracellular uptake, while in cancer cells, this process is markedly amplified, predominantly driven by the enzyme fatty acid synthase (FASN) (19). FASN is a versatile enzyme complex capable of transforming acetyl-CoA and malonyl-CoA into long-chain fatty acids (20). *De novo* FAS furnishes cancer cells with a plentiful lipid reservoir, which supports membrane formation and signal transmission, thus facilitating cancer initiation and advancement (21, 22). Contemporary studies have demonstrated that silencing the tumor suppressor gene Protein Tyrosine Phosphatase, Receptor Type O (PTPRO) enhances FAS, which, in turn, upregulates Acetyl-CoA Carboxylase 1 (ACC1) expression and advances CRC cell growth and liver metastasis (23). Metabolic-associated fatty liver disease (MAFLD) is defined by excessive lipid buildup within the liver’s microenvironment. In MAFLD, lipid-rich microenvironments activate CRLM cells by increasing FASN expression and enhancing the production of endogenous palmitate esters, thereby facilitating the biosynthesis of *de novo* palmitate esters in malignant cells, leading to Epidermal Growth Factor Receptor (EGFR) palmitoylation, which enables membrane localization and evasion of lysosomal degradation (24). Furthermore, cancer cells meet their metabolic needs not only by amplifying *de novo* FAS but also by boosting fatty acid absorption. Fatty acid transport proteins (FATPs) constitute a group of proteins that facilitate the transmembrane movement of fatty acids and are essential for fatty acid uptake in cancer cells. Cancer cells will upregulate the expression of FATPs (e.g., FATP1 and FATP4), significantly enhancing their capacity for fatty acid uptake. Fatty acids are converted to acetyl-CoA, which can enter the β-oxidation pathway or be utilized for phosphatidylcholine synthesis, furnishing cancer cells with bioenergetics and synthetic substrates to sustain their rapid proliferation and metastasis (25). Lipid metabolic reprogramming is pivotal in the formation of PMN. Recent studies have shown that cancer-derived exosomal HSPC111 enhances CRLM by reprogramming lipid metabolism in cancer-associated fibroblasts (CAFs). HSPC111 modifies CAF lipid metabolism through the phosphorylation of ATP-citrate lyase (ACLY), which in turn elevates acetyl-CoA levels. This accumulation of acetyl-CoA promotes the expression and secretion of CXCL5 in CAFs by increasing H3K27 acetylation (26). Consequently, this process facilitates the formation of pre-metastatic niches and supports the establishment of metastatic colonies within the liver microenvironment.

#### 2.1.2 Alterations in fatty acid oxidation

Alterations in fatty acid oxidation (FAO) are one of the key pathways of cellular energy metabolism, primarily carried out through the β-oxidation process in both mitochondria and peroxisomes (27). During the process of liver metastasis, metabolic reprogramming in cancer cells serves a critical function, with FAO alterations notably highlighted (28). Multiple malignant tumors are characterized by the significant overexpression of FAO enzymes Carnitine Palmitoyltransferase 1A (CPT1A), Carnitine Palmitoyltransferase 1B (CPT1B), and Carnitine Palmitoyltransferase-2 (CPT2) (29, 30). Previous research has shown that CRC cells promote FAO by accumulating in adipose tissue-rich tissue and absorbing fatty acids (31). Cancer generates substantial Adenosine Triphosphate (ATP) during metabolic stress and utilizes Nicotinamide Adenine Dinucleotide Phosphate (NADPH), produced by FAO, to neutralize oxidative stress (32). FAO serves a dual function in ensuring cancer cell survival, which is essential. On one hand, cancer cells inhibit β-oxidation of fatty acids in mitochondria, reducing energy production while conserving more fatty acids for biosynthesis. On the other hand, cancer cells activate FAO pathways in peroxisomes, generating substantial reactive oxygen species (ROS), which function as signaling molecules to modulate cancer cell proliferation, migration, and invasion (19). Recent research has shown that ROS play dual roles in cancer cells: at low concentrations, ROS function as signaling molecules to enhance malignant cell proliferation and migration; however, at high concentrations, they lead to cellular damage and apoptosis. During the process of CRLM, cancer cells activate the FAO pathway in peroxisomes to produce an appropriate amount of ROS, thereby promoting their proliferation and invasion (33). Furthermore, Dysregulation of fatty acid oxidation plays a critical role in PMN formation. Metabolic reprogramming of myeloid-derived suppressor cells (MDSCs) through enhanced fatty acid synthesis and mitochondrial oxidative phosphorylation enables them to sustain their immunosuppressive functions within the pre-metastatic liver microenvironment. MDSCs rely on fatty acid synthesis to preserve their immunosuppressive activity (34). Additionally, they contribute to the depletion of arginine in the microenvironment by secreting reactive oxygen species and arginase 1 (ARG1), thereby creating a conducive environment for the colonization of circulating tumor cells (34).

### 2.2 Glucose metabolism reprogramming

Aberrant glucose metabolism is a hallmark of cancer progression, playing a pivotal role in the development and metastatic potential of CRC, particularly in liver metastasis. The establishment of pre-metastatic niches in the liver is critically shaped by the reprogramming of glucose metabolism (12). Primary colorectal tumors release metabolites such as lactate and pyruvate, along with a variety of growth factors. These circulating signals reach the liver and drive coordinated shifts in glucose metabolism within hepatic cells. This conditioning process is marked by the upregulation of glycolytic enzymes and glucose transporters. The outcome is a microenvironment primed for high glucose utilization, which in turn provides an energetic and biosynthetic advantage for incoming metastatic cells. Tumor cells enhance glycolysis (known as the Warburg effect), preferentially converting glucose to lactate even under aerobic conditions to meet the heightened demands for energy and biosynthetic precursors during rapid proliferation (35). The formation of the pre-metastatic niche is closely linked to the induction of enhanced glycolytic capacity within liver cells, a process that occurs prior to the arrival of circulating tumor cells. This metabolic adaptation generates a highly active microenvironment, one that is primed to sustain subsequent colonization. Primary tumor–derived factors play a central role in this reprogramming. They stimulate the expression of key glycolytic enzymes, including hexokinase 2 (HK2), phosphofructokinase (PFK), and lactate dehydrogenase A (LDHA), across multiple hepatic cell types (36). The result is the establishment of a robust metabolic framework. Once tumor cells reach the liver, they can exploit this infrastructure to support their survival, proliferation, and eventual expansion. In CRLM, the glucose-rich hepatic microenvironment exacerbates metabolic reprogramming, not only providing ample metabolic substrates for metastatic tumor cells but also facilitating remodeling of the liver microenvironment (8). Pre-metastatic conditioning reshapes the hepatic microenvironment by establishing glucose gradients and localized zones of lactate accumulation. These alterations act as metabolic cues that can be sensed by circulating tumor cells. Such gradients do more than simply guide the cells toward favorable sites of colonization. They also provide immediate energetic and biosynthetic support once the cells arrive. This dual function facilitates a rapid metabolic adaptation to the hepatic niche and promotes efficient early colonization (37). Dysregulated glucose metabolism also interacts with other metabolic pathways, such as lipid and amino acid metabolism, forming an intricate network that supports tumor progression and adaptation. Consequently, elucidating the underlying mechanisms of glucose metabolism dysregulation in CRLM is critical for identifying novel therapeutic targets and developing strategies to disrupt the metabolic dependencies of metastatic tumors.

#### 2.2.1 Enhanced aerobic glycolysis

Aerobic glycolysis is significantly enhanced and is a hallmark of cancer cell metabolic reprogramming, as first described by Otto Warburg in the 1920s, hence also recognized as the Warburg effect (35, 38). In healthy cells, glucose is mainly metabolized via the oxidative phosphorylation pathway, generating a substantial amount of ATP. However, in tumor cells, glucose is mainly metabolized via the glycolytic pathway even under conditions of sufficient oxygen supply, generating lactate and a small amount of ATP (39). During CRLM, cancer cells enhance aerobic glycolysis to rapidly generate a large amount of ATP, meeting their high energy demands. Although the glycolytic pathway is less efficient, its speed is significantly faster than the oxidative phosphorylation pathway, enabling rapid energy supply (35, 40). Moreover, cancer cells are also known to produce lactic acid and other metabolic products that subdued the pH of the local environment, thereby promoting invasiveness and metastatic activity (41). The acidic microenvironment suppresses the function of immune cells (e.g., T cells and natural killer cells), thereby aiding malignant cells in evading immune surveillance (42). Additionally, it activates enzymes (such as matrix metalloproteinase B and D) in cancer cells, facilitating the degradation of the extracellular matrix (ECM) and thus enhancing the invasive and metastatic potential of malignant cells (43). Furthermore, recent research indicates that lactate not just serves as a metabolic product but also acts as a signaling molecule, regulating malignant cell proliferation, migration, and invasion. For instance, lactate can activate the Hypoxia-Inducible Factor-1α (HIF-1α) signaling pathway, boosting angiogenesis and tumor metastasis (44). Glucose metabolic reprogramming is crucial for the establishment of PMN. The Glut3-YAP signaling pathway serves as a key regulator, driving cancer metabolism and enhancing metastasis. Activation of this pathway increases glucose uptake and glycolytic activity, supplying both energy and metabolic intermediates essential for niche formation. This reprogramming not only supports the survival and growth of cancer cells but also reshapes the hepatic microenvironment, creating conditions conducive to metastatic colonization (45). Additionally, the increased glycolysis within the pre-metastatic niche leads to lactate accumulation, which activates hepatic stellate cells. This, in turn, stimulates the secretion of extracellular matrix components, further establishing a supportive environment for incoming tumor cells (45).

#### 2.2.2 Activation of the hexosamine biosynthetic pathway

The hexosamine biosynthetic pathway (HBP) is a key metabolic branch of glycolysis, responsible for producing uridine diphosphate-N-acetylglucosamine (UDP-GlcNAc), which serves as the donor substrate for protein glycosylation (46). Approximately 2-5% of cellular glucose is diverted from glycolysis into this pathway, which involves a series of enzymatic reactions utilizing glucose, glutamine, acetyl-CoA, and UTP as substrates. The process begins with the conversion of fructose-6-phosphate to glucosamine-6-phosphate, catalyzed by glutamine: fructose-6-phosphate amidotransferase 1 (GFAT1). Several subsequent enzymatic steps lead to the synthesis of UDP-GlcNAc (47). This pathway is essential, as UDP-GlcNAc is the universal donor for O-linked β-N-acetylglucosamine (O-GlcNAc) modifications, making HBP the exclusive source of substrates for this crucial post-translational modification.

The UDP-GlcNAc pathway is a division of sugar metabolism that converts glucose, glutamine, and acetyl-CoA into UDP-GlcNAc, which subsequently participates in protein glycosylation. This glycosylation is a transient and modifiable adjustment occurring after translation that regulates protein function and stability (48). Moreover, the activation of the UDP-GlcNAc pathway is controlled by multiple signaling pathways, including the Phosphatidylinositol 3-Kinase (PI3K)/Protein Kinase B (AKT)/Mammalian Target of Rapamycin (mTOR) pathway, HIF-1α, and Myelocytomatosis Oncogene (MYC). These signaling pathways upregulate key enzymes of the UDP-GlcNAc pathway, such as GFAT1 and O-GlcNAc Transferase (OGT), thereby promoting the generation of UDP-GlcNAc and protein glycosylation (49). During CRLM, the activation of the UDP-GlcNAc pathway enhances glycosylation modifications of multiple proteins, including transcription factors, signaling proteins, and metabolic enzymes among others. These modifications enhance the stability and function of proteins, thereby promoting cancer cell proliferation and metastasis (50). Research has revealed that O-GlcNAc modification enhances AKT activity, promoting tumor cell proliferation and survival (51); O-GlcNAc modification strengthens the stability of HIF-1α, promoting tumor cell angiogenesis and metastasis (52). Furthermore, the activation of the UDP-GlcNAc pathway modulates O-GlcNAc modification to inhibit apoptosis and autophagy in malignant cells, aiding tumor cells in resisting various stress factors during metastasis and enhancing their survival capacity. As an illustration, O-GlcNAc modification suppresses the activity of pro-apoptotic proteins like Bcl-2-Associated Death Promoter (BAD) and Bcl-2-Associated X Protein (BAX), thereby increasing cancer cell survival (53). Activation of the hexosamine biosynthetic pathway plays a pivotal role in PMN formation by influencing protein glycosylation and cellular signaling (47). The activation of the UDP-GlcNAc pathway enhances O-GlcNAc modifications on key transcription factors and signaling proteins in both tumor and stromal cells within the pre-metastatic liver microenvironment. These modifications regulate the expression of genes involved in ECM remodeling, angiogenesis, and immune suppression—factors critical for creating a supportive environment for metastatic tumor cell colonization and growth (54). Furthermore, the metabolic interplay between circulating tumor cells and the hepatic microenvironment via hexosamine pathway activation is a key mechanism in establishing organ-specific metastasis (55).

### 2.3 Amino acid dysmetabolism

Dysregulated amino acid metabolism plays a significant role in the onset and progression of CRLM, with amino acids serving as essential components for protein synthesis, energy production, and the generation of signaling molecules that modulate tumor cell behavior. Pre-metastatic niche formation in the liver also hinges on reprogramming of amino acid metabolism that occurs before tumor cells arrive. Primary colorectal tumors release amino acid metabolites, enzymes, and transport proteins into the circulation. These factors reach the liver and rewire amino acid handling in resident cells. They increase the availability of particular amino acids that support niche formation (56). In CRLM, the liver’s abundant amino acid metabolic pool provides metastatic tumor cells with critical nitrogen sources and biosynthetic precursors, supporting their rapid proliferation and invasion (57). Pre-metastatic conditioning augments hepatic amino acid metabolism. It builds concentrated amino acid pools. It also upregulates amino acid transporters and exchangers. These adaptations provide immediate metabolic support to incoming tumor cells (58). Tumor cells in CRLM exhibit pronounced alterations in amino acid metabolism, primarily involving changes in glutamine metabolism and branched-chain amino acid dysregulation. Disrupted amino acid metabolism also influences the tumor microenvironment in the liver, where competition for amino acids between tumor cells and stromal cells can lead to immunosuppression and enhanced metastatic colonization (59). During pre-metastatic niche formation, niche-forming hepatic cells compete with resident immune cells for amino acids. This early metabolic rivalry establishes immunosuppressive conditions before any tumor cells arrive. Depletion of key amino acids in immune populations blunts antigen presentation and effector function. It weakens cytotoxic activity and limits cytokine production. These deficits precondition the liver for immune escape (60). When tumor cells colonize, they encounter an environment already permissive to evasion and outgrowth. Furthermore, metabolic crosstalk between tumor cells and the hepatic microenvironment, mediated by altered amino acid flux, promotes angiogenesis, inflammation, and extracellular matrix remodeling—all of which are critical for metastatic progression. Thus, elucidating the complex interactions of amino acid metabolism dysregulation in CRLM not only uncovers underlying mechanisms of metastasis but also highlights potential therapeutic targets for disrupting the metabolic dependencies of metastatic tumors.

#### 2.3.1 Glutamine metabolism alteration

Metabolic reprogramming is crucially important during tumor progression and metastasis. Among these adaptations, abnormal glucose and glutamine metabolism is the frequently observed metabolic change in CRC (61). In contrast to the rapid utilization of glucose, glutamine is vital for supporting the tricarboxylic acid (TCA) cycle and acts as a critical precursor for the biosynthesis of fatty acids, nucleotides, and UDP-GlcNAc, respectively. This multifaceted role underscores the importance of glutamine in supporting various biosynthetic and metabolic processes necessary in supporting the growth and survival of malignant cells (62). Research has shown that in CRC tissue, glutamic acid and its downstream metabolites are significantly increased, indicating abnormal activation of glutamine metabolism in CRC cells (63). Cancer cells enhance their glutamine uptake by expressing glutamine transport proteins such as Alanine-Serine-Cysteine Transporter 2 (ASCT2) and System A Sodium-Coupled Neutral Amino Acid Transporter 2 (SN2). Subsequently, glutamine is broken down by glutaminase (GLS) into glutamic acid and ammonia. Glutamic acid is further converted into α-ketoglutaric acid (α-KG), which then enters the TCA cycle. This process provides cancer cells with energy and biosynthetic precursors, supporting their growth and survival (64). Moreover, ammonia produced from glutamine metabolism can regulate intracellular pH and osmotic pressure, thereby enhancing tumor cell proliferation and survival. As an illustration, ammonia can neutralize the acidic environment within tumor cells, thereby enhancing cellular metabolic activity and invasive ability (65). Glutamine metabolism regulates cancer cells’ energy metabolism and signal transduction, promoting its invasion and metastasis. Glutamine metabolism activates the Mechanistic Target of Rapamycin Complex 1 (mTORC1) signaling pathway, stimulating cancer cell proliferation and migration (66). Furthermore, several research have displayed that glutamine depletion in the microenvironment markedly enhances the stem-like characteristics of various cancer cells, contributing to disease progression (67, 68). These outcomes suggest that metabolic variations in tumor cells influence not just intercellular nutrient metabolism but also intrinsic stem cell properties. Glutamine metabolism plays a crucial role in the formation and maintenance of PMN. Changes in glutamine availability within the organ-specific microenvironment shape metabolic preferences and facilitate metastatic colonization. In the pre-metastatic liver, competition for glutamine between tumor and immune cells creates an immunosuppressive environment. CAFs enhance the expression of glutamine synthetase (GLUL), enabling the synthesis of glutamine. This glutamine is transported to pre-metastatic sites via solute carriers, where it activates mTORC1 signaling and boosts the metabolic adaptation of incoming tumor cells (69). Additionally, thrombopoietin (TPO) produced by the liver promotes metastasis of CD110+ tumor-initiating cells by upregulating lysine catabolism, which generates glutamate essential for liver colonization, establishing metabolic dependencies that support metastatic growth (70).

#### 2.3.2 Abnormal metabolism of branched-chain amino acids

Branched-chain amino acids (BCAAs), including phenylalanine, tyrosine, and valine, are crucial amino acids for the human system and are primarily obtained from the diet. In normal cells, BCAAs are primarily metabolized by the branched-chain aminotransferases (BCATs) and the branched-chain α-keto acid dehydrogenase complex (BCKDH), generating the corresponding ketone acids and acetyl-CoA, which enter the TCA cycle to create bioenergetics. This metabolism supports the rapid proliferation and metastasis of malignant cells (71). The metabolic products of BCAAs are not just involved in energy metabolism but function as signaling molecules to modulate the proliferation and migration of malignant cells. The regulation of BCAA metabolism is linked to multiple signaling pathways, including mTORC1 and AMP-activated protein kinase (AMPK). These signaling pathways exert their regulatory effects by modulating the activities of BCAT and BCKDH, thereby influencing the metabolism of BCAAs (72, 73). Furthermore, BCAA metabolism is tied to the regulation of tumor cell epigenetics, impacting tumor cell metabolic reprogramming and metabolic perception (74). BCAA metabolism not only occurs within malignant cells but interacts with various cells within TME. Research has demonstrated that CAFs in the CRC microenvironment undergo alterations in BCAA metabolism, releasing fatty acids and other metabolites to promote tumor cell growth and metastasis (74). Furthermore, BCAA metabolism alterations also impact T cells within the TME, promoting immune evasion (75). Alterations in BCAA metabolism play a critical role in pre-metastatic niche formation by enabling metabolic interactions between tumor cells and the surrounding microenvironment. BCAA metabolism is not limited to tumor cells but also involves extensive crosstalk with various cell types in the tumor microenvironment. In the pre-metastatic liver, CAFs undergo metabolic changes in BCAA pathways, releasing metabolites that support tumor cell colonization and survival (76). Additionally, these metabolic shifts impact T cell function, promoting immune evasion and creating an immunosuppressive environment that aids in the establishment of metastases. BCAA metabolism also influences epigenetic modifications in both tumor and stromal cells, regulating the expression of genes essential for the formation of PMN and successful metastatic colonization (77).

### 2.4 Energy metabolism imbalance

Energy metabolism imbalance is a fundamental characteristic of cancer cells and a critical metabolic feature driving the initiation and progression of CRLM. Pre-metastatic niche formation rests on a deliberate rewiring of hepatic energy metabolism that begins before tumor cells arrive. Primary colorectal tumors release metabolic enzymes, energy metabolites, and signaling molecules into the circulation. They coordinate a shift in core pathways. Glycolysis, oxidative phosphorylation, and fatty acid oxidation are rebalanced to favor high flux and rapid adaptability. The result is an energetically permissive state with broad metabolic flexibility that supports subsequent colonization (78). Insulin resistance, chronic inflammation, and mitochondrial dysfunction disrupt cellular energy sensing and stress responses. They skew AMPK–mTOR signaling and alter redox control. These systemic pressures deepen energy imbalance in the liver. Together, they accelerate pre-metastatic niche establishment (79). Under normal physiological conditions, cells maintain homeostasis by balancing glycolysis, oxidative phosphorylation, and fatty acid oxidation to meet energy demands. However, tumor cells exhibit profound disruptions in energy metabolism to support rapid proliferation, survival, and adaptation to metastatic microenvironments (22). Pre-metastatic conditioning reproduces cancer-like energy disruptions in liver cells. Glycolysis is elevated, oxidative phosphorylation is reshaped, and lipid catabolism is redirected. The result is an energetic state that mirrors tumor cell metabolism. This mimicry gives arriving cancer cells a decisive advantage. They enter a microenvironment already aligned with their own metabolic wiring (78). In CRLM, the glucose-rich hepatic microenvironment and metabolic stress amplify the Warburg effect, where tumor cells preferentially generate ATP and lactate via glycolysis, even in the presence of oxygen, supplying intermediates for nucleotide and lipid synthesis to fuel aggressive behaviors. Pre-metastatic niche formation increases glycolytic capacity in hepatic cells and builds lactate-rich microenvironments. These changes reproduce the Warburg-like conditions that tumor cells favor (80). When metastatic cells arrive, they find glycolytic pathways already primed. Intermediates and biosynthetic precursors are abundant. This imbalance in energy metabolism supplies the resources needed for rapid proliferation and drives aggressive colonization. Additionally, energy metabolism imbalance contributes to an immunosuppressive and pro-tumorigenic microenvironment by promoting lactate accumulation, altering pH, and inducing oxidative stress. Pre-metastatic conditioning disrupts hepatic energy homeostasis. Elevated glycolytic flux drives lactate accumulation and tissue acidosis. Controlled oxidative stress emerges as redox balance shifts. These changes suppress antitumor immunity and prime endothelial activation, promoting angiogenesis before any cancer cells arrive (80). This metabolic program creates an immunosuppressive, pro-tumorigenic niche. It lowers immune surveillance thresholds and weakens barrier functions. It also provides immediate metabolic support. As a result, tumor cells evade immunity and survive from the moment colonization begins. These changes enhance tumor cell survival, suppress anti-tumor immune responses, and facilitate immune escape and metastatic colonization. Understanding energy metabolism imbalance not only elucidates the molecular basis of metabolic reprogramming in CRLM but also identifies potential therapeutic targets for combined strategies targeting glycolysis and oxidative phosphorylation, offering significant prospects for clinical translation.

#### 2.4.1 Mitochondrial dysfunction

Mitochondrial dysfunction is a critical metabolic mechanism contributing towards the development of CRLM. During the metastatic process, cancer cells commonly exhibit mutations in mitochondrial DNA (mtDNA) and damage to the electron transport chain (ETC), driving reduced efficiency of oxidative phosphorylation (OXPHOS). To meet their energy demands, tumor cells shift toward aerobic glycolysis (Warburg effect), achieving this by increasing glucose uptake and lactate production to sustain ATP supply (81). Studies have demonstrated that mitochondrial complex I activity is significantly reduced in metastatic liver lesions, while key glycolytic enzymes such as HK2 and LDHA are upregulated (82, 83). Furthermore, mitochondrial dysfunction induces excessive accumulation of ROS, thereby activating the HIF-1α signaling pathway, which further promotes glycolysis and tumor cell invasion. This metabolic reprogramming provides energy for CRC cells along with supporting the biosynthesis of large molecules through the production of intermediary metabolites like acetyl-CoA, meeting the demand for rapid proliferation (84).

Mitochondrial dysfunction exerts its important role in CRLM by inhibiting cell apoptosis and remodeling the TME. Abnormal permeability of the mitochondrial outer membrane permeabilization (MOMP) facilitates reduced cytochrome C release, thereby inhibiting the caspase-dependent apoptosis pathway and enhancing tumor cell survival capacity (85). The study revealed that the presence of anti-apoptotic proteins B-cell lymphoma 2 (Bcl-2) and Myeloid Cell Leukemia 1 (Mcl-1) is markedly upregulated in the tumor tissue of CRLM patients and correlates with a reduction in mitochondrial membrane potential (ΔΨm) (86, 87). Mitochondrial dysfunction plays a pivotal role in the formation of pre-metastatic niches through the release of damage-associated molecular patterns (DAMPs). In both circulating tumor cells and stromal cells, impaired mitochondria release mtDNA and ATP. These molecules act as signaling agents, activating hepatic stellate cells and tumor-associated macrophages within the liver microenvironment. This activation contributes to the development of an immunosuppressive microenvironment that supports tumor cell colonization (88). Additionally, metabolic stress signals from dysfunctional mitochondria trigger the secretion of pro-inflammatory cytokines, such as IL-6 and TGF-β. These cytokines further enhance pre-metastatic niche formation by remodeling the hepatic microenvironment and inhibiting anti-tumor immune responses (89).

#### 2.4.2 The activation of energy sensors

AMPK and mTOR are central to the metabolic regulation of CRLM. As an energy sensor, AMPK is activated upon ATP depletion, inhibiting the mTORC1 signaling pathway to reduce protein synthesis and cell proliferation, thereby maintaining energy balance (90). In CRLM cells, despite the suppression of AMPK activity and persistent activation of mTORC1 signaling, there is an enhancement of glycolysis and lipid synthesis (91). This metabolic adjustment enables tumor cells to satisfy their high energy requirements by increasing the production of ATP through glycolysis and lipid metabolism, thereby supporting their rapid growth and expansion (92). Research reveals that in the tumor tissue of CRLM patients, AMPKα1 expression is significantly reduced, while the phosphorylation levels of mTORC1 downstream effectors are elevated, and this is correlated with an unfavorable prognosis (93). The AMPK-mTOR signaling axis contributes to regulating autophagy and mitochondrial biogenesis, thereby influencing the adaptability of CRC cells within the metastatic microenvironment (94).

The energy sensor AMPK not only regulates tumor cell metabolism but also remodels the PMN to promote CRLM. AMPK activation inhibits glycolysis and lactate production in CAFs, thereby reducing the “lactate shuttle” effect in the TME, thereby inhibiting CRC cell invasion (95). Conversely, excessive activation of mTORC1 upregulates the abundance of Vascular Endothelial Growth Factor (VEGF) and IL-6 in HSCs, promoting angiogenesis and the formation of an immune-suppressive microenvironment (96). Furthermore, energy sensors modulate the metabolic interaction between tumor cells as well as hepatocytes, influencing the formation of metastatic lesions. Studies have demonstrated that AMPK activation inhibits CRC cells from taking up glutamine, reducing α-KG production, which inhibits the behavior of chromatin-modifying enzymes namely Ten-Eleven Translocation 2 (TET2), thereby repressing the levels of metastasis-related genes (97). These findings indicate that energy sensors have a multifaceted impact on the metabolic regulation of CRLM.

### 2.5 Metabolic remodeling of the microenvironment

Microenvironmental metabolic reprogramming is a pivotal mechanism driving the onset and progression of CRLM, with the TME—comprising tumor cells, stromal cells, immune cells, and metabolites—exerting profound regulatory effects on CRLM’s invasive and metastatic potential (98). The formation of pre-metastatic niches marks the initial step in the metabolic remodeling of the microenvironment, driven by primary tumor-derived factors. These factors systematically reprogram the liver’s metabolic landscape, preparing it for tumor cell colonization before any cancer cells arrive. The liver, as a highly metabolic organ, provides a uniquely nutrient-rich niche that metastatic tumor cells exploit to establish and thrive. However, the presence of tumor cells induces significant metabolic alterations within the hepatic microenvironment, fostering reciprocal interactions among cancer cells, stromal cells, immune cells, and endothelial cells. The transition from a pre-metastatic niche to an active metastatic microenvironment is driven by the integration of arriving tumor cells into pre-existing metabolic networks. The metabolic framework established during pre-metastatic conditioning offers immediate support to tumor cells while facilitating ongoing remodeling of the microenvironment. This process promotes the co-evolution of cancer cells and stromal cells, ensuring the persistence and progression of metastasis (80). This metabolic crosstalk drives the reprogramming of glucose, lipid, and amino acid metabolism, which not only sustains the energy demands of tumor cells but also reshapes the TME into a pro-tumorigenic and immunosuppressive niche. Furthermore, metabolic remodeling in the TME impairs immune cell function, suppressing anti-tumor immunity and promoting immune evasion (99). The metabolic remodeling initiated during pre-metastatic conditioning lays the groundwork for sustained immune suppression and metabolic dysfunction, which intensify following tumor cell colonization. The pre-existing metabolic changes in immune cells impair their anti-tumor activity, fostering an environment that supports tumor immune evasion and ongoing growth. These dynamic changes underscore the active role of the hepatic microenvironment in supporting CRLM and highlight the therapeutic potential of targeting metabolic reprogramming. Thus, elucidating the mechanisms of intercellular interactions in microenvironmental metabolic remodeling not only enhances understanding of the metabolic regulatory networks in CRLM but also offers significant clinical and translational prospects for developing combined therapeutic strategies targeting TME metabolism.

#### 2.5.1 Metabolic alterations in tumor-associated fibroblasts

CAFs in CRLM contribute to tumor cell energy and metabolic precursor requirements through metabolic reprogramming. Research has demonstrated that CAFs within the TME exhibit a significant “inverse Warburg effect,” characterized by enhanced OXPHOS. Simultaneously, through high glycolytic activity, CAFs release extensive amounts of lactate, ketones, and amino acids (such as glutamine) into the microenvironment, directly supporting CRC cells’ energy needs (100). In the CRLM model, CAFs secrete lactate via the monocarboxylic acid transporter Monocarboxylate Transporter 4 (MCT4). This lactate is subsequently absorbed by tumor cells via Monocarboxylate Transporter 1 (MCT1) and utilized for oxidative metabolism, facilitating their proliferation and invasion (101). Furthermore, CAFs, which upregulate GLUL to synthesize glutamine, transport this glutamine to tumor cells through the solute carrier, thereby activating the mTORC1 signaling pathway to enhance protein synthesis (102). This metabolic symbiosis significantly enhances tumor cell survival capacity in the hypoxic and nutrient-deprived metastatic microenvironment.

The metabolic products of CAFs not only serve as energy substrates but can also act through paracrine signals to remodel the pre-metastatic liver microenvironment. CAFs secrete lactate, which activates G Protein-Coupled Receptor 81 (GPR81) on tumor cell surfaces. This activation induces HIF-1α stabilization, resulting in the upregulation of angiogenic factors like VEGF and matrix metalloproteinases (MMP2/9), promoting blood vessel formation and extracellular matrix breakdown (103, 104). Simultaneously, CAFs release ketones, such as β-hydroxybutyric acid, which inhibit histone deacetylases in hepatocytes. This inhibition induces epigenetic activation of metastasis-promoting genes like Snail Family Transcriptional Repressor 1 (SNAIL) and Twist Family BHLH Transcription Factor 1 (TWIST), thus promoting the colonization of CRC cells in the liver (105). Further clinical and pathological analysis demonstrates that CAFs in CRLM nodules exhibit abnormal lipid metabolism, leading to increased prostaglandin E2 (PGE2) secretion. This is mediated through Prostaglandin E Receptor 4 (EP4) receptor signaling, which suppresses CD8+ T cell functionality and promotes regulatory T cell infiltration, thereby forming an immunosuppressive microenvironment (106).

#### 2.5.2 Metabolic reprogramming in immune cells

The development of CRLM relies not only on the invasive and metastatic capacity of malignant cells but on alterations in the liver microenvironment. Among these, metabolic reprogramming in immune cells plays a crucial element in modulating the liver metastasis process. Metabolic reprogramming refers to immune cells reorganizing their metabolic pathways in a specific TME to adapt to new survival needs. This process directly influences the functional status of immune cells and thereby shapes the onset and advancement of liver metastasis. Metabolic changes in the TME occur through the reshaping of immune cell metabolic pathways, inhibiting their anti-tumor function. For example, tumor cells consume glucose and glutamine through competitive consumption, which block the metabolism of immune cells and thereby inhibit their function (107). Furthermore, the buildup of metabolic products like lactic acid and guanidine acid in immunosuppressive regions further exacerbates the functional impairment of immune cells (108).

In CRLM, metabolic reprogramming in immune cells is characterized by alterations in the metabolic pathways of various immune cells. First, metabolic reprogramming in T cells directly influences its differentiation, function, and anti-tumor efficacy. In the CRLM microenvironment, CD8+ T cells shift toward FAO to maintain survival due to competition for glucose, but this is accompanied by a marked reduction in their functional capabilities, such as the secretion of Interferon-γ (IFN-γ) (109). Studies have demonstrated that the expression of the key glycolysis enzyme Pyruvate Kinase M2 (PKM2) in tumor-infiltrating T cells (TILs) is downregulated, leading to metabolic paralysis and exhaustion of the cells (110). While regulatory T cells (Tregs) achieve selective expansion in a low-glucose environment by upregulating GLS and enhancing OXPHOS, they inhibit anti-tumor immunity (111). In clinical intervention, Programmed Death-1 (PD-1) inhibitors restore the glycolytic capacity of CD8+ T cells, thereby reversing exhaustion, but their efficacy is limited by tumor lactate levels (108). Moreover, metabolic reprogramming in TAMs determines their prometastatic or anti-tumor phenotype. M1-type macrophages rely on glycolysis and the pentose phosphate pathway (PPP) to generate ROS to damage tumor cells. However, TAMs in CRLM sites often polarize into M2-type macrophages, enhancing OXPHOS and lipid synthesis (e.g., cholesterol esterification), thereby secreting Interleukin-10 (IL-10) and TGF-β to promote immune suppression (112). Metabolomic studies have revealed that in the liver metastasis microenvironment, an imbalance in arginine metabolism (upregulated ARG1) inhibits M1 polarization and induces macrophages to produce polyamines via the urea cycle, thereby accelerating tumor cell invasion. Targeting TAMs’ metabolic nodes, such as inhibiting ARG1 or blocking fatty acid uptake, can restore their anti-tumor function and inhibit liver metastasis (113). Finally, MDSCs also establish an immunosuppressive microenvironment in CRLM through unique metabolic adaptability. MDSCs depend on FAS and mitochondrial OXPHOS to maintain their immunosuppressive functions, and they further deplete arginine in the microenvironment by secreting ROS and ARG1 (114). Preclinical research have shown that inhibiting MDSCs can markedly decrease liver metastasis burden and enhance the synergistic effect of anti- Programmed Death-Ligand 1 (PD-L1) therapy (115).

Recent years have seen the emergence of targeting metabolic reprogramming in immune cells as a novel approach for treating CRLM. By intervening in key metabolic nodes, such as inhibiting LDHA or indole 2,3-dioxygenase (IDO1), it is possible to boost the anti-tumor activity of immune cells (108). Furthermore, research has also discovered that metabolites from gut microbiota, like short-chain fatty acids (SCFAs), can be achieved through epigenetic modification to reshape T cell differentiation, thereby further influencing the immune response (116). These studies have established a theoretical foundation for novel immunotherapy approaches and introduced new strategies to counter immune resistance (107). Future research should further investigate the clinical application potential of these metabolic intervention strategies to improve treatment outcomes in CRLM.

Immune cell metabolic reprogramming plays a critical role in pre-metastatic niche formation, preparing distant organs for metastatic colonization. In the pre-metastatic liver, metabolic changes in resident immune cells create an immunosuppressive environment prior to tumor cell arrival. Kupffer cells and hepatic macrophages undergo shifts that promote M2 polarization, marked by increased oxidative phosphorylation and lipid synthesis. These reprogrammed macrophages secrete immunosuppressive cytokines, such as IL-10 and TGF-β, while depleting arginine through heightened arginase activity. This creates an environment that suppresses anti-tumor immunity, facilitating tumor cell colonization (117). Additionally, natural killer T (NKT) cells in the pre-metastatic liver exhibit altered metabolic profiles that reduce their cytotoxic function, further fostering an immune-privileged microenvironment that supports metastatic growth. Tumor-derived factors, including exosomes and metabolites, often trigger this metabolic reprogramming, underscoring the systemic nature of pre-metastatic niche formation (118).

## 3 Mechanisms underlying the core components of metabolic syndrome in driving colorectal cancer liver metastasis

Metabolic syndrome, characterized by insulin resistance and encompassing obesity, diabetes, hypertension, and hyperlipidemia, is increasingly recognized as a critical factor in the progression and metastatic spread of CRC, particularly to the liver. CRLM represents a major clinical challenge and is a leading cause of mortality in CRC patients. Emerging evidence suggests that the core components of MS not only exacerbate systemic metabolic dysfunction but also foster a tumor-promoting microenvironment that enhances the initiation, growth, and metastatic colonization of CRC cells in the liver (3). Consequently, systematically dissecting the driving mechanisms of MS components in CRLM not only elucidates the interplay between metabolic dysregulation and tumor metastasis but also provides a theoretical foundation for developing precision therapeutic strategies targeting MS-related pathways (**Table 1**).

**Table 1:** Mechanistic links between core components of metabolic syndrome and colorectal cancer liver metastasis.

Components of metabolic syndrome	Classification of mechanisms	Core process	Key molecules/pathways	Functional impact	Experimental models	Clinical implications
Obesity	Adipose tissue inflammation	M1 polarization of macrophages and secretion of pro-inflammatory factors (TNF-α, IL-6)	TNF-α/NF-κB, IL-6/JAK2-STAT3	Promotion of tumor cell survival, invasion, and hepatic colonization	High-fat diet mouse model; *In vitro* inflammatory factor stimulation assay	Serum TNF-α levels as a predictive biomarker for liver metastasis risk
	Adipokine dysregulation	Leptiin↑, adiponectin↓	Leptiin/JAK2-STAT3, adiponectin/AMPK	Leptin promotes tumor proliferation and metastasis; adiponectin inhibits tumor growth	Serum analysis of obese patients; leptin receptor knockout mouse model	The leptin/adiponectin ratio is significantly associated with patient prognosis
	Insulin resistance	Hyperinsulinemia activates pro-proliferative signaling	IGF-1/PI3K-Akt, MAPK	Enhancement of tumor cell survival and metastatic potential	Diabetic mouse model; *In vitro* high-insulin stimulation assay	IGF-1 levels are positively correlated with the number of liver metastases
	Dysregulation of lipid metabolism	Elevated levels of free fatty acids (FFA) and Cholesterol	FFA/TLR4, SREBP2/LDLR	Promotion of tumor cell invasion and hepatic colonization	High-cholesterol diet mouse model; lipidomics analysis	Serum cholesterol levels can be used to assess the risk of liver metastasis
Diabetes Mellitus	Hyperinsulinemia	Activation of the insulin/IGF-1→PI3K-Akt/mTOR pathway	IGF-1/PI3K-Akt and MAPK pathways	Promotion of tumor cell survival, proliferation and metastasis; inhibition of apoptosis	Hyperinsulinemic mouse model; *In vitro* insulin stimulation assay	Serum IGF-1 levels are positively correlated with the number of liver metastases
	Chronic inflammation	Increased secretion of inflammatory factors (TNF-α, IL-6) and activation of inflammatory signaling pathways	TNF-α/NF-κB, IL-6/JAK2-STAT3 pathways	Inflammatory factors promote immunosuppression in the tumor microenvironment and enhance tumor cell invasion and hepatic colonization capacity	Diabetic mouse model; inflammatory factor detection assay	Serum TNF-α and IL-6 levels are positively correlated with the risk of liver metastasis
	Oxidative stress	Accumulation of ROS leads to DNA damage and genomic instability	ROS/NF-κB, HIF-1α/VEGF pathways	Promotion of tumor cell malignant transformation and metastasis	Detection of oxidative stress markers; antioxidant intervention assay	Oxidative stress markers (e.g., 8-OHdG) can be used to assess the risk of liver metastasis
	Extracellular matrix (ECM) remodeling	High expression of matrix metalloproteinases (MMPs) promotes ECM degradation	High expression of MMP2/MMP9; TGF-β/Smad pathway	Promotion of tumor cell epithelial-mesenchymal transition (EMT) and enhancement of invasive and metastatic potential	In vitro matrigel invasion assay; diabetic mouse model	Serum MMP9 levels are positively correlated with the risk of liver metastasis
	Angiogenesis	Hyperglycemia and hyperinsulinemia promote the secretion of angiogenic factors (VEGF, FGF)	VEGF/VEGFR, FGF/FGFR pathway	Promotion of tumor angiogenesis to provide nutrients and metastatic pathways for tumor cells	Angiogenesis assay; diabetic mouse model	VEGF levels can be used to evaluate the efficacy of anti-angiogenic therapy
Hyperlipidemia	Reprogramming of lipid metabolism	Tumor cells depend on lipid synthesis to meet energy demands	High expression of fatty acid synthase (FASN) and stearoyl-CoA desaturase-1 (SCD-1)	Promotion of tumor cell proliferation, invasion and metastasis	High-fat diet mouse model; lipidomics analysis	Serum fatty acid levels can serve as predictive biomarkers for the risk of liver metastasis
	Cholesterol accumulation	Cholesterol accumulation enhances tumor cell membrane fluidity	Regulation by LDLR/SREBP2; downregulation of ABCA1	Promotion of tumor cell invasion and metastasis	High-cholesterol diet mouse model; cholesterol metabolism assay	Serum cholesterol levels are positively correlated with the risk of liver metastasis
	Role of FFA	FFA activate inflammatory signaling pathways	FFA/TLR4-NF-κB pathway	Induction of inflammatory responses promotes immunosuppression and metastasis within the tumor microenvironment	FFA stimulation experiment; toll-like receptor 4 (TLR4) knockout mouse model	Serum free fatty acids levels can be used to assess the risk of liver metastasis
	Oxidative stress	The accumulation of ROS leads to DNA damage and genomic instability	ROS/NF-κB, HIF-1α/VEGF pathway	Facilitating the malignant transformation and metastasis of tumor cells	Detection of oxidative stress markers; antioxidant intervention experiment	Oxidative stress markers can be used to assess the risk of liver metastasis
	Inflammatory microenvironment	Increased secretion of inflammatory factors (TNF-α, IL-6) and activation of inflammatory signaling pathways	TNF-α/NF-κB, IL-6/JAK2-STAT3 pathway	Inflammatory factors promote immunosuppression in the tumor microenvironment and enhance tumor cell invasion and hepatic colonization capacity	High-fat diet mouse model; inflammatory cytokine detection assay	Serum levels of TNF-α and IL-6 are positively correlated with the risk of hepatic metastasis
Hypertension	The role of Angiotensin II (Ang II)	Ang II promotes tumor cell invasion and metastasis by activating downstream signaling pathways through the Angiotensin II type 1 receptor (AT1R)	Ang II/AT1R → MMP2/MMP9; TGF-β/Smad	Promoting EMT, thereby enhancing the invasive and metastatic capabilities of tumor cells	Hypertensive rat model; Ang II stimulation experiment	Serum levels of Ang II are positively correlated with the risk of hepatic metastasis
	Angiogenesis	Hypertension promotes the secretion of angiogenic factors, such as VEGF and fibroblast growth factor (FGF)	VEGF/VEGFR, FGF/FGFR	Promoting tumor angiogenesis, thereby providing nutrients and metastatic pathways for tumor cells	Angiogenesis assay; hypertensive mouse model	VEGF levels can be utilized to evaluate the efficacy of anti-angiogenic therapy
	Hemodynamic alterations	elevated blood pressure leads to increased vascular shear stress, promoting tumor cell colonization in the liver	Hemodynamics-related molecules (e.g., Shear Stress Sensors)	Enhancing the intrahepatic colonization ability of tumor cells	Hemodynamic simulation assay; hypertensive mouse model	Blood pressure control may reduce the risk of liver metastasis
	ECM Remodeling	Hypertension promotes high expression of MMPs and degradation of the ECM	MMP2/MMP9; TGF-β/Smad	Promotion of tumor cell EMT and enhancement of invasive and metastatic potential	*In vitro* matrigel invasion assay; hypertensive mouse model	Serum MMP9 levels are positively correlated with the risk of liver metastasis
	Adrenergic receptor stimulation	Activation of β-Adrenergic Receptors (β-AR)	Activation of β-AR/cAMP/PKA signaling pathway	Promotion of tumor cell proliferation, invasion, and metastasis; inhibition of immune cell activity	*In vitro* β-AR agonist (e.g., isoproterenol) stimulation experiment	β-AR antagonists (e.g., Propranolol) may serve as a novel strategy for treating liver metastasis

### 3.1 Obesity and colorectal cancer liver metastasis

It is widely recognized that obesity is linked to the development of various cancers. Obesity is considered a pivotal triggering factor for the maturation of CRC. The relationship between obesity and CRC has been confirmed by numerous studies. Epidemiological studies suggest that individuals with obesity are at a significantly higher risk of developing CRC than those with normal weight (119). Increasing evidence indicates that persistent inflammation elicited by obesity results in the loss of insulin pathway and internal balance control mechanisms, serving as the mechanism linking obesity to primary CRC (120). Metabolic disorders related to obesity, namely insulin resistance, chronic inflammation, and lipid metabolism abnormalities, create a conducive microenvironment for the development and advancement of CRC (121). MAFLD is caused by high-fat diet and obesity. It encompasses all metabolic fatty liver diseases from steatosis to fatty liver hepatitis. It represents the liver aspect of metabolic syndrome. MAFLD is linked to hepatocellular carcinoma (HCC) and CRLM. This indicates that the special metabolic environment of fatty liver is conducive to tumor establishment and development (122). Systemic obesity may enhance the likelihood of developing primary CRC, and, via its impact on the liver, may also indirectly result in the progression of CRLM in these patients (123).

Obesity, as an essential part of metabolic syndrome, drives the onset and advancement of CRLM through multiple pathophysiological mechanisms. Obesity-induced adipose tissue dysfunction leads to systemic chronic low-level inflammation, where adipocytes and CAFs release abundant pro-inflammatory factors (e.g., IL-6, Tumor Necrosis Factor-α (TNF-α)) and adipokines (e.g., leptin, adiponectin), thereby remodeling the TME and facilitating pre-metastatic niche formation. Elevated leptin levels directly enhance the proliferative, invasive, and migratory capacities of CRC cells through activation of the Janus Kinase 2 (JAK2)/Signal Transducer and Activator of Transcription 3 (STAT3) and PI3K/AKT signaling pathways, while simultaneously suppressing adiponectin expression, exacerbating insulin resistance, and disrupting metabolic homeostasis (124). Obesity-associated hyperactivation of the insulin/Insulin-like Growth Factor 1 (IGF-1) signaling pathway further facilitates tumor cell energy metabolic reprogramming and protein synthesis through the mTORC1 pathway, thereby providing the bioenergetic foundation for liver metastasis (125). Furthermore, obesity-induced lipid metabolic dysregulation significantly elevates circulating free fatty acid (FFA) levels. These fatty acids, upon uptake by tumor cells in hepatic metastases, not only provide energy through β-oxidation but also stimulate the nuclear receptor Peroxisome Proliferator-Activated Receptor-γ (PPARγ), thereby regulating the representation of pro-metastatic genes (126). Notably, obesity-associated MAFLD provides a ‘fertile soil’ for liver metastasis: ROS and pro-inflammatory mediators released by steatotic hepatocytes disrupt the completeness of the liver sinusoidal endothelial barrier, while simultaneously recruiting immunosuppressive cells (e.g., M2 macrophages and regulatory T cells), thereby creating an immune-privileged microenvironment that facilitates metastatic tumor cells’ evasion of immune surveillance (127). Clinical explorations have further indicated that obese patients exhibit a significantly elevated incidence of liver metastasis relative to non-obese individuals, with roughly a 40% higher risk of postoperative recurrence, indicating that obesity acts as a standalone predictor for CRLM (128). These mechanisms collectively constitute a complex network through which obesity drives CRLM, thereby providing a scientific rationale for intervention strategies targeting lipid metabolism and inflammatory pathways.

### 3.2 Diabetes mellitus and colorectal cancer liver metastasis

The pathological correlation between diabetes mellitus and CRLM stems from the synergistic effects of its characteristic metabolic dysregulation and microenvironment remodeling. Chronic hyperglycemia induces ‘metabolic memory’ in tumor cells, persistently activating glycolysis and mitochondrial respiratory chain-induced oxidative stress, leading to excessive accumulation of ROS. This subsequently impairs Deoxyribonucleic Acid (DNA) repair mechanisms and stimulates the HIF-1α signaling pathway, thereby promoting the abundance of metastasis-associated genes (e.g., Lysyl Oxidase (LOX), Matrix Metalloproteinase-2 (MMP-2), VEGF), which accelerates extracellular matrix degradation and angiogenesis (129, 130). Concurrently, compensatory hyperinsulinemia induced by insulin resistance drives abnormal tumor cell growth and resistance to apoptosis through the PI3K/AKT/mTORC1 axis, while overproduction of IGF-1 further initiates the Mitogen-Activated Protein Kinase (MAPK) pathway, thereby enhancing tumor cells’ adaptability to the hepatic microenvironment (5). The characteristic chronic mild inflammatory condition in diabetes mellitus releases pro-inflammatory factors such as IL-6 and TNF-α through Nuclear Factor Kappa B (NF-κB)-dependent pathways, which not only directly stimulate the invasive phenotype of malignant cells but also recruit M2 macrophages and Tregs, thereby establishing an immunosuppressive microenvironment that diminishes the anti-tumor action of CD8+ T cells (131). Clinical investigations have demonstrated that the frequency of liver metastasis is 27% elevated in diabetic patients in comparison to non-diabetic individuals, with significantly enhanced chemoresistance in metastatic lesions, which may be linked to hyperglycemia-induced enrichment of cancer stem cells and autophagy activation (132, 133). Notably, diabetes-associated vascular complications-induced hepatic hypoxia and fibrosis promote hepatic stellate cell activation through the TGF-β/Smad pathway, leading to the secretion of laminin and collagen, thereby constructing a ‘pre-metastatic niche’ conducive to tumor cell colonization (134). This multi-level pathological network suggests that targeting glucose metabolic reprogramming (e.g., Sodium-Glucose Co-Transporter 2 (SGLT2) inhibitors) or modulating the inflammatory microenvironment (e.g., anti-IL-6 antibodies) may represent potential strategies for improving liver metastasis prognosis in diabetic patients.

The hyperglycemic state in diabetic patients also provides an abundant energy source for CRC cell growth. Under hyperglycemic conditions, tumor cells enhance the glycolytic pathway, generating increased energy and metabolic intermediates to support their rapid proliferation and metastasis (135). Furthermore, diabetes facilitates the progression of CRLM by affecting hepatic metabolic function. Research has demonstrated that diabetic patients exhibit increased hepatic fat accumulation, leading to fatty liver disease. This alteration in the hepatic microenvironment provides supportive conditions for tumor cell growth and metastasis (136). Diabetes interacts with other components of metabolic syndrome (e.g., obesity, hypertension), further exacerbating the probability of CRLM. For instance, the combination of obesity and diabetes significantly amplifies both the incidence of CRLM (137, 138).

### 3.3 Hyperlipidemia and colorectal cancer liver metastasis

Hyperlipidemia is widely acknowledged as a primary contributor to atherosclerosis and coronary heart disease. The accumulation of cholesterol in the bloodstream can lead to the development of atherosclerotic plaques, representing one of the main drivers of coronary heart disease (139). Over the last few years, numerous investigations have identified a contact between abnormal lipid levels and tumor development. Clinically, several epidemiological studies have demonstrated that lifestyle choices leading to hyperlipidemia, such as a high-fat diet (HFD), can stimulate CRC progression (140). Clinical evidence indicates that statins may reduce the incidence of CRC-related mortality (141). Another study demonstrated that both hyperlipidemia and hypercholesterolemia serve as standalone risk factors for CRLM. In this study, patients with liver metastasis exhibited markedly elevated levels of total cholesterol (TC) and triglycerides (TG) compared to the non-metastatic group. Notably, the proportion of hyperlipidemic patients was notably above in the liver metastasis group, suggesting that dyslipidemia may promote CRLM through multiple mechanisms (125). Therefore, it is warranted to investigate the mechanisms through which hyperlipidemia influences CRC metastasis.

Hyperlipidemia, as a key component of metabolic syndrome, directly drives the biological processes of CRLM through dysregulated lipid metabolism. Excess circulating cholesterol and triglycerides are taken up by tumor cells via low-density lipoprotein receptor (LDLR) or scavenger receptor Scavenger Receptor Class B Type 1 (SR-B1), thereby activating the PI3K/AKT/mTOR signaling pathway and boosting tumor cell proliferation and anti-apoptotic capabilities (142). Cholesterol metabolic intermediates, such as oxysterols (e.g., 27-hydroxycholesterol), induce epithelial-mesenchymal transition (EMT) in CRC cells by binding to estrogen receptor α (ERα) or liver X receptor (LXR), thereby enhancing their invasiveness and promoting hepatic metastatic colonization (143). Simultaneously, FFAs released from hypertriglyceridemia activate the PPARγ/δ pathway through membrane receptor CD36, regulating FAO and energy metabolic reprogramming, thereby providing sustained energy supply for metastatic lesions (144). Furthermore, hyperlipidemia-induced oxidative stress triggers the production of ROS, impairing DNA repair mechanisms and activating the NF-κB signaling pathway, thereby exacerbating tumor genomic instability and promoting the expression of metastasis-associated genes (145).

Hyperlipidemia also creates a ‘fertile soil’ for CRLM by remodeling the hepatic microenvironment. Chronic hyperlipidemia promotes hepatic lipid accumulation, leading to MAFLD. Steatotic hepatocytes release pro-inflammatory factors (e.g., IL-6, TNF-α) and chemokines (e.g., Chemokine Ligand 2 (CCL2)), recruiting MDSCs and M2 macrophages, thereby constructing an immunosuppressive microenvironment (127). Lipid peroxidation products, like 4-hydroxynonenal (4-HNE), activate the Hedgehog signaling pathway, inducing HSC activation and fibrosis, thereby forming a collagen- and laminin-rich pre-metastatic niche (134). Clinical cohort research has shown that patients with hyperlipidemia exhibit a 40% elevated risk of CRLM compared to individuals with normal lipid levels, along with significantly reduced chemotherapy response rates in metastatic lesions. These findings may be associated with lipid metabolism-related enrichment of cancer stem cells and autophagy activation (125). These mechanisms suggest that targeting key nodes in lipid metabolism (e.g., inhibiting CD36 or LXR) may represent a novel strategy for intervening in liver metastasis.

### 3.4 Hypertension and colorectal cancer liver metastasis

Hypertension promotes CRLM through systemic hemodynamic disturbance and vascular endothelial dysfunction in the early stage. Research indicates that the increased permeability of hepatic sinusoidal endothelial cells in hypertensive patients facilitates cellular transendothelial migration, enabling tumor cells to more easily traverse vascular barriers and establish metastasis in the liver. The higher likelihood of liver metastasis in hypertensive patients may be associated with the upregulation of VEGF (146). Furthermore, the local hypoxic environment induced by hypertension activates the HIF-1α signaling pathway, promoting the establishment of the liver pre-metastatic microenvironment. This is manifested by the activation of liver sinusoidal cells and restructuring of the extracellular matrix, which provides support for CRC cell adhesion and proliferation (147, 148).

According to related reports, hypertension significantly increases the risk of developing malignant tumors, and it serves as a crucial risk factor that contributes to the death of the tumor population (149, 150). Adrenergic receptor stimulation is linked to the progression of hypertension (HTN) and is also tied to cancer progression and the dissemination of various tumors (including CRC) (151). Beta-blocker drugs (BBS), such as beta-adrenergic antagonists, exhibit an inhibitory effect on the invasion and migration of CRC cells and are indicative of a decrease in the death rate of CRC (152, 153). Retrospective analysis indicates that patients taking renin-angiotensin system (RAS) inhibitors experience a decrease in CRC incidence, reduced polyp formation, and a decrease in distant metastasis (154).

The core pathophysiological mechanism of hypertension—activation of the RAS—directly regulates molecular processes involved in CRLM. Animal experiments demonstrate that angiotensin II (Ang II) activates NF-κB and STAT3 signaling pathways by binding to AT1 receptors (AT1R), inducing CRC cell EMT and enhancing their infiltrative and migratory abilities (155). Clinical pathological analysis further confirms that Ang II levels are dramatically upregulated in the liver metastatic nodules of hypertensive patients, and this exhibits a significant positive association with the high expression of MMP-9 within these nodules, suggesting that Ang II facilitates tumor cell extravasation by degrading the basement membrane (156). Furthermore, RAS signals regulate the M2 polarization of liver Kupffer cells, inhibiting the anti-tumor immune response and forming an immunosuppressive microenvironment, thereby further accelerating the metastatic process (157).

### 3.5 Synergistic mechanisms of metabolic syndrome components in driving colorectal cancer liver metastasis

Metabolic syndrome in clinical practice is characterized by the concurrent presence of several metabolic abnormalities that interact in a synergistic manner, creating conditions that promote metastasis to a much greater extent than would be expected from the individual effects of each abnormality. These interactions often involve overlapping molecular pathways, common signaling hubs, and interconnected metabolic disturbances, all of which collectively drive the progression of CRLM (**Table 2**). Understanding these complex, synergistic mechanisms is crucial for developing effective therapeutic strategies. Such strategies must target the multifaceted nature of MS-driven CRLM, rather than focusing solely on isolated metabolic abnormalities.

**Table 2:** Synergistic mechanisms between metabolic syndrome components in driving colorectal cancer liver metastasis.

MS Component Combinations	Classification of synergistic mechanisms	Core synergistic process	Key molecules/pathways	Functional impact	Clinical implications
Obesity + Diabetes	Metabolic-inflammatory convergence	Convergent activation of PI3K/AKT/mTOR and JAK2/STAT3 pathways with amplified inflammatory response	Hyperinsulinemia + leptin → mTOR convergence; AGEs + adipokines → NF-κB VEGF	Enhanced tumor cell invasive capacity; systemic inflammation; lipid-rich microenvironment creation	CRLM risk increased; requires dual metabolic intervention
Obesity + Hypertension	Hemodynamic-adipokine interaction	Leptin-enhanced membrane fluidity synergizes with Ang II-induced vascular remodeling	Leptin/JAK2-STAT3 + Ang II/AT1R → MMP activation; enhanced endothelial permeability	Optimized tumor cell extravasation; vascular barrier disruption; oxidative stress	Liver metastasis rate ↑vs individual conditions; vascular protection needed
Obesity + Hyperlipidemia	Lipid metabolism maximization	Dual lipid supply through synthesis (leptin→FASN) and uptake (cholesterol→LDLR/SR-B1)	Leptin → STAT3/PI3K + cholesterol → mTOR; lipid droplets; ATP production	Maximal energy substrate availability; membrane synthesis support; inflammatory amplification	Strongest lipid metabolic synergism; dual lipid pathway targeting required
Diabetes + Hypertension	Vascular-glycemic synergism	AGEs-induced endothelial damage amplified by Ang II-mediated vascular stress	AGEs/RAGE + Ang II/AT1R → NF-κB/MAPK; endothelial permeability; VCAM-1	Enhanced vascular permeability; angiogenic synergism; glucose metabolic reprogramming	Liver vascular density ↑; dual vascular-metabolic protection needed
Diabetes + Hyperlipidemia	Lipid-glucose metabolic cycle	Insulin resistance-lipid accumulation vicious cycle with dual metabolic pathway activation	Hyperinsulinemia → SREBP-1c + FFA → insulin resistance; ATP production	Maximal metabolic substrate availability; enhanced cell death resistance; chemotherapy resistance	Dual metabolic addiction; multi-pathway metabolic targeting required
Hypertension + Hyperlipidemia	Vascular-lipid interaction	Oxidized LDL endothelial damage synergizes with pressure-induced mechanical stress	Oxidized LDL/LOX-1 + mechanical stress → endothelial permeability; MMP activation	Optimal extravasation conditions; vascular remodeling; severe oxidative stress	Maximal vascular dysfunction; comprehensive vascular intervention needed
Obesity + Diabetes + Hyperlipidemia	Complete metabolic activation	Simultaneous activation of all major metabolic pathways with maximal inflammatory response	Triple convergence: insulin/IGF-1→PI3K/AKT + leptin→JAK2/STAT3 + cholesterol→mTOR; ATP	Maximal growth signaling; systemic inflammation	CRLM risk↑; requires comprehensive metabolic intervention
Obesity + Diabetes + Hypertension	Vascular-metabolic maximization	Triple vascular assault: adipokine + AGEs + mechanical stress on endothelium	Leptin + AGEs + Ang II → maximal endothelial dysfunction; vascular resistance, permeability	Maximal vascular compromise; systemic oxidative stress; optimal metastatic conditions	CRLM risk ↑; multi-system vascular protection required
Complete Metabolic Syndrome (All 4 components)	Systemic metabolic dysregulation	Complete disruption of metabolic homeostasis with "inflammatory storm"	All major pathways activated: PI3K/AKT/mTOR, NF-κB, JAK/STAT, HIF-1α	Maximum CRLM promotion; 203 metabolites altered across 12 pathways; CTCs	CRLM risk ↑; requires intensive multi-target therapy; poor prognosis marker

#### 3.5.1 Obesity-diabetes synergistic mechanisms

The coexistence of obesity and diabetes is one of the most clinically relevant synergistic drivers in MS-related CRLM. Obesity-induced insulin resistance aggravates hyperglycemia. At the same time, the chronic inflammatory state associated with diabetes further impairs adipose tissue function (158). Together, these processes form a self-perpetuating cycle of metabolic disruption, creating a pathological environment that strongly favors disease progression.

At the molecular level, the coexistence of obesity and diabetes drives a synergistic activation of the PI3K/AKT/mTOR signaling axis through multiple converging mechanisms. Hyperinsulinemia in diabetes stimulates insulin receptor activity, while obesity-related hyperleptinemia activates JAK2/STAT3-mediated growth signaling. These parallel cascades intersect at the mTOR complex, amplifying protein synthesis, cell proliferation, and anti-apoptotic responses well beyond the impact of either condition alone (159). Studies have shown that CRC cells exposed to elevated insulin and leptin demonstrate an increase in invasive potential compared with single-agent exposure. Clinical evidence mirrors these findings (160). Patients with concurrent obesity and diabetes exhibit a markedly higher risk of CRLM. In contrast, obesity or diabetes alone confer more modest risks (161).

The synergistic inflammatory response constitutes a pivotal mechanism in the interplay between obesity and diabetes. In obese diabetic individuals, adipose tissue macrophages display heightened M1 polarization, accompanied by elevated secretion of pro-inflammatory mediators such as TNF-α, IL-6, and IL-1β. At the same time, hyperglycemia promotes the formation of advanced glycation end products (AGEs), which activate hepatic Kupffer cells via RAGE signaling. This dual process amplifies systemic inflammation and fosters the development of a PMN. The cumulative inflammatory load drives persistent NF-κB activation and stimulates the release of angiogenic factors, thereby creating a microenvironment favorable for tumor colonization (162). Mechanistic studies further reveal that the combined influence of AGEs and adipokines enhances NF-κB p65 phosphorylation and upregulates VEGF expression, underscoring the multiplicative impact of these inflammatory signals (163).

Metabolically, the coexistence of obesity and diabetes induces profound dysregulation of lipid homeostasis through synergistic alterations in fatty acid synthesis and oxidation. Hyperinsulinemia markedly upregulates FASN and ACC1 expression in hepatocytes as well as in tumor cells. In parallel, obesity-related hyperleptinemia increases FATP expression, thereby enhancing lipid uptake. These changes generate an abundance of lipid substrates that not only drive accelerated tumor cell proliferation but also promote hepatic steatosis (164). The result is a lipid-enriched microenvironment that facilitates metastatic colonization. Metabolic flux analyses further confirm this synergy, showing an increase in palmitic acid synthesis and a rise in lipid uptake within the liver of obese diabetic patients (165).

#### 3.5.2 Obesity-hypertension synergistic effects

The coexistence of obesity and hypertension produces distinctive synergistic effects driven by the interplay between RAS activation and adipokine signaling. This interaction exerts complementary influences on vascular integrity and cellular metabolism (166). As a result, tumor cells acquire greater capacity for extravasation and more efficient hepatic colonization, thereby accelerating metastatic progression.

Obesity-related leptin elevation acts in concert with hypertension-driven Ang II, producing a potent synergistic effect. Leptin stimulates tumor proliferation and epithelial–mesenchymal transition via JAK2/STAT3 signaling. At the same time, Ang II activates NF-κB and STAT3 through AT1 receptors, leading to increased MMP expression and extracellular matrix degradation (167). This cooperation operates on multiple levels. Leptin enhances membrane fluidity, thereby facilitating MMP secretion and enzymatic activity. In parallel, Ang II promotes vascular remodeling, generating favorable conditions for tumor cell extravasation (167). Clinical observations reinforce these mechanistic insights, showing that patients with both obesity and hypertension have a higher incidence of liver metastasis compared with those affected by either condition alone (168).

Vascular dysfunction emerges as a key synergistic mechanism in the context of obesity and hypertension. Imbalances in adipokines associated with obesity impair hepatic sinusoidal endothelial cells, leading to heightened permeability. Concurrently, elevated blood pressure exerts mechanical stress that facilitates tumor cell transmigration (169). Experimental models further highlight this interaction: under combined obese-hypertensive conditions, characterized by high leptin and increased pressure, endothelial permeability rises compared with either factor alone (170).

Oxidative stress synergy plays a particularly destructive role in obesity–hypertension interactions. Adipose tissue–derived ROS converge with ROS produced by hypertension-driven NADPH oxidase activation. The result is an excessive oxidative burden that accelerates DNA damage and promotes genomic instability. Clinical observations support this mechanism, showing that oxidative stress markers such as 8-OHdG rise in obese hypertensive patients compared with those presenting either condition alone (171).

#### 3.5.3 Obesity-hyperlipidemia synergistic mechanisms

Among dual comorbidities, the coexistence of obesity and hyperlipidemia produces the most profound disruption of lipid metabolism. This interaction markedly accelerates CRLM by activating multiple lipid-dependent signaling cascades. The metabolic surplus provides abundant energy substrates for tumor growth. At the same time, it shapes a microenvironment enriched in pro-inflammatory and pro-angiogenic factors, thereby fostering metastatic progression (24).

At the cellular level, elevated leptin promotes lipid uptake in tumor cells by activating STAT3 and PI3K/AKT signaling, which upregulates receptors such as LDLR, SR-B1, and CD36. In the presence of hyperlipidemia, excess circulating lipids are efficiently internalized through these pathways, further stimulating mTOR activation and *de novo* lipid synthesis (172). CRC cells from obese hyperlipidemic patients exhibit striking metabolic changes: lipid droplet accumulation increases, while ATP production rises. These alterations provide both structural substrates and energetic support, directly fueling rapid tumor growth and metastatic progression (173).

The inflammatory synergy between obesity and hyperlipidemia is particularly pronounced. In obesity, activated macrophages release pro-inflammatory mediators, while hyperlipidemia promotes lipid peroxidation, intensifying the inflammatory cascade. Products of lipid peroxidation, such as 4-hydroxynonenal, activate hepatic stellate cells and Kupffer cells. At the same time, leptin directly stimulates these cells to secrete IL-6, TNF-α, and various chemokines (174).

#### 3.5.4 Diabetes-hypertension synergistic effects

The coexistence of diabetes and hypertension is observed in nearly 60% of diabetic patients. This dual condition produces intricate vascular and metabolic synergies, with pronounced effects on hepatic microvasculature and glucose metabolic reprogramming (175). It illustrates how distinct pathophysiological processes can converge, amplifying disease progression and leading to outcomes far more severe than those caused by either disorder alone.

At the molecular level, diabetes and hypertension interact to produce synergistic endothelial injury. Chronic hyperglycemia impairs hepatic sinusoidal endothelial cells through protein glycation and oxidative stress. In parallel, Ang II increases endothelial permeability via AT1 receptor activation. The synergy arises from crosstalk between AGEs binding to RAGE receptors and Ang II engaging AT1 receptors. This dual activation amplifies NF-κB and MAPK signaling, driving inflammatory and structural changes (176).

Angiogenic synergy is another pivotal mechanism in the interaction between diabetes and hypertension. In diabetes, hyperglycemia stabilizes HIF-1α, while hypertension promotes VEGF expression through Ang II–driven AP-1 activation. Together, these processes generate a strongly pro-angiogenic microenvironment. The dual stimulation markedly increases hepatic microvascular density. This vascular expansion ensures a rich nutrient supply, creating favorable conditions for metastatic tumor growth (177).

#### 3.5.5 Diabetes-hyperlipidemia synergistic mechanisms

The coexistence of diabetes and hyperlipidemia generates intricate interactions between glucose and lipid metabolism. Insulin signaling is dysregulated at the same time that lipid metabolic pathways are activated, producing a tightly interwoven network of disturbances (178). This dual burden is frequently observed in patients with metabolic syndrome. Its mutually reinforcing mechanisms not only accelerate disease progression but also create significant challenges for therapeutic intervention.

Insulin resistance and lipid dysregulation form a self-reinforcing cycle. Hyperinsulinemia activates Sterol Regulatory Element-Binding Protein-1c (SREBP-1c), driving fatty acid synthesis. At the same time, elevated circulating lipids worsen insulin resistance, deepening metabolic imbalance. In tumor cells, this cycle provides dual advantages. Insulin-activated PI3K/AKT signaling enhances glucose uptake and glycolysis. Meanwhile, excess lipids supply substrates for β-oxidation and membrane biosynthesis. Together, these processes markedly boost energy availability (179). Study show ATP production in tumor cells from diabetic hyperlipidemic patients rises compared with either condition alone (173).

The inflammatory synergy of diabetes and hyperlipidemia arises from the combined action of AGEs and oxidized lipids. AGEs engage RAGE receptors, while oxidized low density lipoprotein (LDL) activates toll-like receptor 4 (TLR4). These signals converge on NF-κB, triggering extensive release of pro-inflammatory cytokines. A highly tumor-promoting microenvironment characterized by enhanced angiogenesis and suppression of antitumor immunity (180).

Resistance to cell death is a key synergistic outcome of diabetes–hyperlipidemia comorbidity. Elevated insulin activates AKT signaling, reinforcing anti-apoptotic pathways. At the same time, excess cholesterol stabilizes mitochondrial membranes, further suppressing apoptosis. These dual mechanisms provide tumor cells with strong protection against cytotoxic stress. As a result, they become markedly less responsive to chemotherapy and immune-mediated clearance (181).

#### 3.5.6 Hypertension-hyperlipidemia synergistic effects

The coexistence of hypertension and hyperlipidemia produces distinct synergistic effects involving both vascular function and lipid metabolism. These interactions primarily compromise vascular integrity and enhance tumor cell extravasation during metastasis. This dual influence illustrates how separate pathophysiological processes can converge to accelerate defined steps in cancer progression (182).

Endothelial dysfunction stands out as the central synergistic mechanism in hypertension–hyperlipidemia comorbidity. Oxidized LDL directly injures endothelial cells and suppresses nitric oxide production. Meanwhile, mechanical stress from elevated blood pressure further disrupts endothelial integrity. The synergy arises from the interplay between oxidized LDL activating LOX-1 receptors and pressure-induced mechanosensitive ion channel signaling. This dual assault markedly increases vascular permeability (183).

Vascular remodeling synergy is largely mediated by persistent activation of the RAS. In hyperlipidemia, lipid overload stimulates renin secretion, while hypertension sustains RAS activity, leading to excess production of Ang II. Ang II engages AT1 receptors, driving the upregulation of MMP-2 and MMP-9 and accelerating basement membrane degradation. At the same time, oxidized lipids activate NF-κB signaling, which further amplifies MMP expression (184). Together, these processes reshape the vascular niche, creating conditions highly favorable for tumor cell extravasation.

#### 3.5.7 Obesity-diabetes-hyperlipidemia triple synergism

The coexistence of obesity, diabetes, and hyperlipidemia produces the most powerful pro-CRLM synergy. It simultaneously amplifies metabolic disturbances, inflammatory cascades, and growth signaling pathways. This triple burden, often seen in advanced metabolic syndrome, drives a dramatic rise in metastatic risk (185).Metabolic synergy reaches its peak when glucose and lipid pathways are fully activated. Hyperinsulinemia stimulates glucose uptake and accelerates glycolysis. At the same time, leptin promotes lipid uptake, while elevated circulating lipids supply abundant substrates for β-oxidation. Together, these processes converge to generate optimal conditions for energy production and biosynthesis (186).

Signal transduction synergy arises from the concurrent activation of several growth-promoting pathways and their extensive crosstalk. Insulin and IGF-1 stimulate PI3K/AKT signaling. Leptin engages the JAK2/STAT3 axis. Cholesterol further drives mTOR activation. Together, these pathways reinforce one another, generating maximized proliferative signals (187).

#### 3.5.8 Obesity-diabetes-hypertension triple synergism

The coexistence of obesity, diabetes, and hypertension generates highly complex vascular–metabolic synergies. These interactions profoundly alter hepatic hemodynamics and reshape the metabolic microenvironment, thereby facilitating CRLM. Clinical evidence underscores the severity of this combination, showing an increase in CRLM risk compared with patients carrying single conditions (188).

Vascular impairment reaches its peak under the combined burden of obesity, diabetes, and hypertension. Endothelial dysfunction, heightened permeability, and abnormal angiogenesis act together to destabilize vascular homeostasis. In obesity, adipokine imbalance weakens endothelial function. Diabetes adds further injury through AGE-mediated damage. Hypertension contributes mechanical stress that disrupts vascular barriers (189). Overactivation of the RAS represents a central mechanism in the coexistence of obesity, diabetes, and hypertension. In obesity, renin secretion is stimulated. Hypertension sustains chronic RAS activity. Diabetes amplifies the pro-inflammatory effects of Ang II through the AGEs–RAGE axis. Together, these processes drive hepatic fibrosis, vascular remodeling, and persistent inflammation. The resulting microenvironment provides highly favorable conditions for metastatic tumor cell colonization (190).

Oxidative stress becomes systemic under the combined influence of obesity, diabetes, and hypertension. Multiple sources of ROS are activated simultaneously. Adipose tissue macrophages release excess ROS, hyperglycemia amplifies oxidative byproducts of glucose metabolism, and hypertension stimulates NADPH oxidase activity. Together, these mechanisms generate a profound oxidative burden (191).

#### 3.5.9 Complete metabolic syndrome (Quadruple synergistic effects)

The coexistence of obesity, diabetes, hypertension, and hyperlipidemia defines the most severe state of metabolic dysfunction. This complete syndrome produces the strongest pro-metastatic drive for CRLM. Systemic metabolic reprogramming reaches its peak in patients with complete metabolic syndrome. Multiple pathways are activated in parallel, including glycolysis, fatty acid synthesis and oxidation, amino acid metabolism, and nucleotide biosynthesis (56). This broad metabolic activation fuels rapid tumor growth, while also reinforcing stem cell–like properties and resistance to therapy.

The pro-inflammatory state becomes systemic in advanced metabolic syndrome. Multiple inflammatory triggers are activated at once, creating a storm-like environment highly favorable to tumor progression. Such an overwhelming inflammatory burden not only accelerates tumor growth but also impairs anti-tumor immune surveillance, further promoting metastatic spread (192). Vascular and circulatory disturbances reach their highest level in complete metabolic syndrome. Endothelial function is profoundly impaired, vascular permeability markedly elevated, and aberrant angiogenesis becomes widespread. These changes directly enhance metastatic spread (193).

## 4 Metabolic dual axis in CRLM: insulin and adipokine regulation

It is known that each component of metabolic syndrome is associated with tumor development. However, the effects of these components can be promoted through additive or cooperative multiple molecular mechanisms, contributing to the onset and progression of the disease.

### 4.1 Insulin resistance/high insulin levels/insulin-like growth factor-1 system

Insulin resistance is commonly linked to elevated insulin levels and is considered the central component of metabolic syndrome. Insulin, as the primary anabolic metabolic hormone, stimulates cell proliferation, and it is thought to directly stimulate cancer cell proliferation and metastasis (194). Extensive epidemiological evidence shows that diabetes is deemed as a standalone risk element for the higher incidence and mortality of heterogeneous cancers (195). The rates of incidence and mortality for various cancers, including pancreatic, hepatocellular, colon, breast, endometrial, and bladder cancers, have shown a substantial increase in diabetic patients (196–198). The insulin/IGF axis, which includes insulin resistance, high insulin levels, and IGF, along with high blood glucose, inflammatory cytokines, and sex hormones, collectively create a favorable environment for cancer cell (199–201).

Insulin resistance (IR) and hyperinsulinemia are the core features of metabolic syndrome. Through the activation of IR and insulin-like growth factor-1 receptor (IGF-1R) signaling pathways, they facilitate the progression of CRLM (202). IR reduces the susceptibility of peripheral tissues to insulin, driving up insulin levels in the blood. This high insulin level, through feedback mechanisms, further increases insulin production. The PI3K/AKT/mTOR signaling pathway, which is involved in cell proliferation and viability, is activated. This activation promotes cancer cell growth while suppressing apoptosis. Clinical studies have demonstrated that patients with elevated insulin levels face a significantly greater susceptibility to CRLM (HR = 1.56). Additionally, elevated levels of phosphorylated AKT (p-AKT) in tumor tissue are connected to a worse prognosis for these patients (202). Furthermore, high insulin levels boost tumor cell glucose uptake through the upregulation of Glucose Transporter 1 (GLUT1) transport proteins, thereby meeting the energy demands required for their rapid growth (203).

The IGF-1 system is essential for CRLM through paracrine and autocrine mechanisms. IGF-1, secreted by the liver and by CAFs, activates the RAS/MAPK and PI3K/AKT signaling pathways by binding to the IGF-1R, thereby promoting tumor cell invasion and metastasis (204). Animal experiments demonstrate that the knockdown of IGF-1R significantly inhibits the formation of CRLM nodules and is associated with an increase in E-cadherin expression in tumor cells (205). Furthermore, IGF-1 induces the activation of HSCs and remodels the ECM, thereby forming a pre-metastatic microenvironment (206).

Insulin and IGF-1 signaling promote CRLM by regulating immune cell functions in the TME. Hyperinsulinemia activates the PI3K/AKT pathway in tumor cells, upregulating PD-L1 expression and subsequently suppressing the anti-tumor activity of CD8+ T cells (207). Simultaneously, IGF-1 encourages the progression of an immunosuppressive microenvironment by promoting the expansion of Tregs and MDSCs, thereby facilitating tumor cell immune evasion (208). These results demonstrate that insulin/IGF-1 signaling acts as a cornerstone in the immunometabolic regulation of tumor metastasis.

### 4.2 The leptin and adiponectin system

Leptin is a functional protein generated through the transcription and translation of the obesity gene, with its receptors widely distributed throughout the body. Leptin exerts long-term effects on the hypothalamus, suppressing appetite and increasing basal metabolism, thereby regulating food intake and energy balance (209). In contrast to leptin, adiponectin exhibits anti-inflammatory and anti-atherosclerotic properties, promoting insulin sensitization and reducing the incidence of various metabolic disorders (210). Hypoadiponectinemia is linked to insulin resistance, type 2 diabetes, atherosclerosis, coronary heart disease, and the progression of malignant tumors (211). Patients with CRLM typically exhibit decreased serum adiponectin levels and higher plasma concentrations of TNF-α and leptin. This characteristic profile of adipocytokine levels is implicated in the increased incidence of liver metastasis (212). Explorations have confirmed the contact between adiponectin/leptin balance and mitogenesis, tumor growth, and cellular motility processes during adipose tissue dysfunction (213).

Leptin, a hormone secreted by adipocytes, is significantly elevated in metabolic syndrome patients due to obesity and accelerates the progression of CRLM through activation of its receptor (LepR). Upon binding to LepR, leptin activates the JAK2/STAT3 and PI3K/AKT signaling pathways, inducing tumor cell proliferation, EMT, and enhanced invasive capabilities (214). Studies have demonstrated that in CRC, the positive immunostaining rates for STAT3 and p-STAT3 are 72% (36/50) and 76% (38/50), respectively (215). A review of 17 studies involving 2,346 CRC patients demonstrated a direct relationship between p-STAT3 expression and lymph node metastasis (216). Furthermore, leptin stimulates Kupffer cells in the liver to excrete IL-6 and TNF-α, creating a pro-inflammatory microenvironment that facilitates the emergence of a pre-metastatic niche (217, 218).

Adiponectin, a protective hormone secreted by adipocytes, is significantly reduced in metabolic syndrome patients, leading to diminished inhibitory effects on CRLM. Adiponectin activates the AMPK and PPAR-α signaling pathways, inhibiting tumor cell glycolysis and FAS, thereby restricting the energy supply to tumor cells (219, 220). Furthermore, adiponectin reduces the discharge of pro-inflammatory factors (e.g., IL-6 and Monocyte Chemoattractant Protein-1 (MCP-1)) by inhibiting the NF-κB pathway, thereby blocking M2 polarization of TAMs and maintaining an anti-tumor immune microenvironment (221). However, insufficient adiponectin levels in metabolic syndrome patients result in the failure of this protective mechanism.

The imbalance between elevated leptin levels and reduced adiponectin levels, known as ‘leptin-adiponectin axis dysregulation,’ creates a synergistic effect in metabolic syndrome patients, accelerating CRLM. Leptin promotes tumor angiogenesis by upregulating HIF-1α and VEGF (222, 223), Conversely, adiponectin deficiency leads to reduced expression of anti-angiogenic factors (e.g., thrombospondin-1), further exacerbating vascular abnormalities (224). Simultaneously, leptin-induced inflammatory signaling and adiponectin-suppressed AMPK pathway inactivation collectively promote tumor cell metabolic reprogramming, enhancing their adaptability to hypoxic microenvironments (225, 226). Clinical intervention studies have demonstrated that restoring adiponectin levels through exercise or pharmacological agents (e.g., thiazolidinediones) can partially reverse leptin’s pro-metastatic effects and reduce the incidence of CRLM (227).

## 5 Applications of metabolomics in colorectal cancer liver metastasis research

The application of metabolomics in CRLM research has become a key focus in cancer research in recent years. Over the past decade, metabolomics has offered key insights into the molecular mechanisms and clinical translation of CRLM through systematic analysis of dynamic changes in the TME and systemic metabolites (**Table 3**). Studies have demonstrated that gut microbiota-derived metabolites play crucial roles in regulating hepatic immune tolerance and liver regeneration processes (221). Gut microbiota dysbiosis can lead to excessive activation of NKT cells by Kupffer cells in the liver, increasing IFN-γ levels and thereby suppressing liver regeneration (221). Patients with CRLM exhibit significant metabolic reprogramming characteristics in both plasma and tumor tissues, including enhanced glutamine dependency, dysregulated bile acid metabolism, and lactate accumulation (130). Metabolomic analyses utilizing mass spectrometry and nuclear magnetic resonance have revealed that elevated levels of succinate and ketone bodies in liver metastases are associated with oxidative stress and HIF-1α signaling activation, suggesting their potential as metastatic drivers (228). Targeted metabolomics has identified alterations in serum levels of tryptophan metabolites (e.g., kynurenine) and short-chain fatty acids (e.g., butyrate), which can predict liver metastasis risk and differentiate between early and advanced metastasis (229, 230). Clinical integrative analyses have further demonstrated that the combined application of metabolomics and transcriptomics data can identify key regulatory nodes (e.g., ACLY and Isocitrate Dehydrogenase 1 (IDH1)), providing a rationale for combination therapies targeting glycolysis and lipid synthesis (231, 232). These studies have not only revealed the central role of metabolic heterogeneity in tumors but also laid the scientific foundation for developing precision therapeutic strategies based on metabolic intervention.

**Table 3:** Application of metabolomics in the study of colorectal cancer liver metastasis.

Application directions	Technical platforms	Sample types	Key findings	Potential biomarkers	Clinical significance	Study limitations
Early diagnosis	1.Liquid chromatography-mass spectrometry(LC-MS) 2.Nuclear magnetic resonance(NMR) 3.Integrated metabolomics-transcriptomics analysis	Serum/Plasma Feces Primary tumor tissue	1.Significant elevation of tricarboxylic acid (TCA) cycle intermediates (e.g., succinate) in the serum of patients prior to liver metastasis 2.Dysregulation of glutamine metabolism is associated with early metastasis 3.Reduced levels of short-chain fatty acids (SCFAs) in feces	Serum: succinate, glutamine, alanine Feces: butyrate, propionate Tissue: lactate, ketone bodies	1.Non-invasive screening tools to enhance the detection rate of liver metastasis in conjunction with imaging 2.Distinguishing primary cancer from early metastasis	1.High sample heterogeneity (e.g., diet and gut microbiota interference) 2.Lack of large-scale validation cohorts 3.Mechanistic associations remain unclear
Prognostic monitoring	1.Gas chromatography-mass spectrometry(GC-MS) 2.Targeted metabolomics 3.Spatial metabolomics (e.g., MALDI-MSI)	Metastatic lesion tissue Peripheral blood Urine	1.Elevated levels of sphingomyelins (SMs) in liver metastases indicate poor prognosis 2.Abnormal bile acid profiles in blood are associated with the risk of recurrence 3.Polyamine metabolites (e.g., Spermine) in urine are associated with reduced survival	Tissue: sphingomyelins, ceramides Blood: deoxycholic acid, glycocholic acid Urine: spermine, spermidine	1.Dynamic assessment of metastasis progression risk to guide postoperative monitoring frequency 2.Stratifying patients for personalized treatment	1.The dynamic changes in metabolites are complex, necessitating repeated sampling 2.The resolution of spatial metabolomics is currently insufficient 3.The clinical translation process is characterized by a prolonged timeline
Therapeutic response monitoring	1.Ultra-performance liquid chromatography-mass Spectrometry(UHPLC-MS) 2.Metabolic flux analysis(^13^C Labeling)	Paired serum samples before and after treatment Organoid models tumor Microenvironment samples	1.Patients with chemotherapy resistance exhibit elevated serum lactate levels, indicative of enhanced glycolysis 2.In responders to targeted therapy, the fatty acid β-oxidation pathway is suppressed 3.Metabolic reprogramming of aspartate in organoids is associated with oxaliplatin sensitivity	Serum: lactate, pyruvate, carnitine Organoids: aspartate, glutathione Microenvironment: kynurenine	1.Real-time assessment of chemotherapy/targeted therapy efficacy; 2.Predicting drug resistance and adjusting treatment regimens; 3.Developing metabolism-targeting drugs	1.Heterogeneity in treatment regimens compromises data comparability 2.The mechanisms underlying dynamic metabolic responses remain poorly understood 3.Organoid models exhibit significant discrepancies compared to *in vivo* conditions

### 5.1 Applications of metabolomics in early diagnosis of CRC

Metabolomics, through large-scale analysis of small molecule metabolites in biological samples, offers new insights into the early diagnosis of CRC. Studies have demonstrated significant differences in serum, urine, and fecal metabolic profiles between CRC patients and healthy individuals. Research has shown that CRC patients exhibit significantly reduced levels of SCFAs in serum, while levels of amino acids (e.g., glutamine, leucine) and lipid metabolites (e.g., lysophosphatidylcholines) are markedly elevated (233). The alterations in these metabolites reflect the metabolic reprogramming during the early stages of CRC, including enhanced glycolysis, aberrant glutamine metabolism, and increased lipid synthesis. The metabolomics-based biomarker panel (comprising citrate, succinate, and alanine) demonstrates high sensitivity and specificity for differentiating early-stage CRC patients from healthy controls, offering a potential tool for non-invasive diagnosis (234).

The high sensitivity and high-throughput characteristics of metabolomics technologies, namely Liquid Chromatography-Mass Spectrometry (LC-MS), Gas Chromatography-Mass Spectrometry (GC-MS), and Nuclear Magnetic Resonance (NMR), enable the quantification of low-abundance metabolites, thereby revealing potential biomarkers for the early diagnosis of CRC. LC-MS-based metabolomic analysis has identified significantly upregulated levels of bile acids in the feces of CRC patients, which are closely associated with gut microbiota dysbiosi (235). Furthermore, GC-MS analysis has revealed abnormal alterations in TCA cycle intermediates (such as citrate and α-ketoglutarate) in the urine of CRC patients, suggesting the involvement of mitochondrial dysfunction during the initial phases of CRC development (236). These metabolic biomarkers not only facilitate early diagnosis but also hold potential for monitoring the progression and recurrence of CRC.

In spite of the considerable promise of metabolomics in the early diagnosis of CRC, its translation to clinical practice is still hindered. Firstly, the concentration of metabolites is susceptible to influences from diet, medication, and individual variability, necessitating the establishment for standardized protocols in sample collection and analysis (237). Secondly, the complexity and high-dimensional nature of metabolomics data necessitate the development of more efficient bioinformatics tools for data integration and interpretation (238). Future studies should incorporate multi-omics data (such as genomics and proteomics) with machine learning algorithms to construct more accurate early diagnostic models for CRC, thereby advancing the translation of metabolomics from the laboratory to clinical practice.

### 5.2 The potential of metabolomics in prognostic prediction

Metabolomics, through the systematic analysis of metabolic profiles in CRLM patients, offers novel biomarkers and molecular insights for individualized prognostic assessment. Studies have revealed that specific metabolic signatures, such as abnormal accumulation of sphingolipids (e.g., sphingosine-1-phosphate, S1P) and bile acid metabolites (e.g., deoxycholic acid), are present in the plasma and tumor tissues of patients with liver metastasis. These metabolic alterations are significantly associated with the aggressiveness of metastatic lesions and reduced overall survival (OS) in patients (239). These metabolites accelerate the metastatic process by modulating inflammatory responses (e.g., the NF-κB pathway) and angiogenesis (e.g., VEGF signaling) within the TME (240). S1P promotes tumor cell drug resistance by activating the STAT3 pathway, while deoxycholic acid enhances metastatic potential by inducing DNA damage and oxidative stress (241). These findings suggest that metabolomics can reveal the molecular heterogeneity of CRLM and provide a basis for prognostic stratification.

Metabolomics-based models demonstrate high accuracy and clinical translational potential in prognostic prediction. A study utilizing metabolomic analysis revealed the metabolic characteristics of CRC patients. By employing LC-MS to examine the serum metabolic profiles of CRC patients, the study identified Ubiquitin Specific Peptidase 3 Antisense RNA 1 (USP3-AS1) as a novel promoter of CRLM through the induction of histone lactylation. USP3-AS1 was significantly linked to tumor aggressiveness and overall patient survival (241). Integrating metabolomics with radiomics data can further enhance prediction accuracy. A study employing a machine learning (ML) model combined with the analysis of volatile organic metabolites in exhaled breath from 62 patients after radical CRC resection demonstrated promising sensitivity and specificity in predicting the development of CRLM or local recurrence (242).

Future research needs to address the challenges of metabolomics in prognostic prediction. The clinical translation of metabolic biomarkers is currently limited by sample heterogeneity and insufficient standardization in detection, necessitating the establishment of multicenter validation cohorts and unified analytical protocols (243). Furthermore, multi-omics integration strategies represent a critical direction for future research. By combining metabolomics, genomics, proteomics, and other multi-omics data, a more comprehensive understanding of disease mechanisms can be achieved, resulting in the identification of more accurate prognostic biomarkers. For instance, the SIMO tool, which integrates spatial multi-omics data, enables the combination of diverse omics data with spatial information, thereby uncovering intercellular regulatory relationships and spatial regulatory patterns (244). This integrative approach facilitates the characterization of metabolic networks and control processes mechanisms closely associated with disease prognosis, thereby providing stronger support for personalized medicine.

### 5.3 The application of metabolomics-revealed biomarkers associated with metabolic syndrome in colorectal cancer liver metastasis

Metabolic syndrome (MetS) is strongly linked to the pathological mechanisms of CRLM. In recent years, metabolomics technologies have enabled researchers to gradually uncover the critical role of MetS-related metabolic dysregulation in the initiation and progression of liver metastasis, proposing biomarkers with diagnostic and prognostic value. By enabling high-throughput analysis of metabolites in biological samples, metabolomics has also provided new perspectives on early diagnosis and prognostic prediction of CRLM.

Metabolomics studies have revealed the significant roles of various biomarkers associated with metabolic syndrome in CRLM. For instance, alterations in lipid metabolites exhibit notable differences in CRLM. Research has identified that certain lipid metabolites, such as lysophosphatidylcholine (LPC) and S1P, are markedly increased in the plasma of CRC patients (245, 246). These alterations in metabolites not only reflect the metabolic reprogramming of malignant cells but are closely associated with changes in the TME. Furthermore, metabolic syndrome-related metabolites, such as the lactate-to-pyruvate ratio, have been shown to be associated with the prognosis of CRLM. An elevated lactate-to-pyruvate ratio is linked to tumor hypoxia and enhanced glycolysis, which may promote tumor invasion and metastasis (247).

The combined application of metabolomics and multi-omics technologies has further enhanced the understanding of CRLM. By integrating metabolomics, genomics, and proteomics data, we can more comprehensively decipher the metabolic characteristics and regulatory mechanisms of tumors. A study utilizing LC-MS for untargeted metabolomic analysis combined with transcriptomics compared frozen liver metastasis biobank samples from RCC and LCC patients. The metabolomics results revealed an increase in ROS-related metabolites and bile acids in RCC. In contrast, carnitine (an indicator of FAO) was relatively increased in LCC. Transcriptomic analysis demonstrated enhanced MEK-ERK, PI3K-AKT, and TGF-β signaling in RCC liver metastasis. The differences revealed by the integration of metabolomics and transcriptomics may be associated with the clinical disparities in tumor behavior between RCC and LCC liver metastases (248). Furthermore, the integrated analysis of metabolomics and genomics has unveiled associations between metabolic syndrome-related gene mutations and alterations in metabolites, offering new targets for personalized treatment (247).

Despite significant progress in revealing MetS-related biomarkers through metabolomics, its clinical application still faces challenges. Firstly, the concentration of metabolites is susceptible to influences from diet, medication, and individual variability, necessitating the establishment for standardized protocols in sample collection and analysis (243). Secondly, the complexity and high-dimensional nature of metabolomics data require the development of more efficient bioinformatics tools for data integration and interpretation (249). Future studies should combine multi-omics data (such as genomics and proteomics) with artificial intelligence technologies to construct more accurate predictive models for CRLM and explore therapeutic strategies targeting MetS-related metabolic pathways (250).

## 6 Prospects

Although considerable progress has been achieved in elucidating the mechanisms linking metabolic syndrome to CRLM, future research still requires in-depth exploration in several areas. Firstly, further advancements in metabolomics technologies will provide more precise tools for clarifying the role of metabolites related to metabolic syndrome in the process of CRLM. Secondly, multi-omics integration strategies (such as the combined analysis of metabolomics, genomics, and proteomics) hold promise for uncovering core metabolic pathways and molecular networks driving CRLM, offering new targets for precision medicine. Additionally, the clinical translation of metabolic syndrome-related metabolites still faces challenges related to standardization and individual variability. Establishing multicenter, large-scale metabolomics databases and integrating artificial intelligence technologies (such as deep learning models) can optimize the screening and validation processes of metabolic biomarkers, enhancing their accuracy in early diagnosis and prognostic prediction. Future research should also explore personalized treatment strategies guided by metabolomics, such as combining targeted therapies for metabolic syndrome-related metabolic pathways with immune checkpoint inhibitors, which may offer more effective therapeutic choices for CRLM patients. Lastly, research on metabolic syndrome and CRLM should emphasize interdisciplinary collaboration and the conduct of international multicenter clinical trials. By integrating basic research, clinical data, and real-world evidence, a comprehensive research chain from mechanistic exploration to clinical application can be established, promoting the transition of metabolomics from the lab to the clinic. It is believed that through metabolomics analysis and in-depth research on metabolic syndrome, we can discover novel therapeutic targets and design personalized treatment strategies for CRLM patients, potentially improving clinical outcomes and patient prognosis.
